# *De novo* sequencing of the *Hypericum perforatum* L. flower transcriptome to identify potential genes that are related to plant reproduction *sensu lato*

**DOI:** 10.1186/s12864-015-1439-y

**Published:** 2015-03-31

**Authors:** Giulio Galla, Heiko Vogel, Timothy F Sharbel, Gianni Barcaccia

**Affiliations:** Laboratory of Plant Genetics and Genomics, DAFNAE – University of Padova, Campus of Agripolis, Viale dell’Università 16, 35020 Legnaro, Italy; Max Planck Institute for Chemical Ecology, Hans-Knöll-Straße 8, 07745 Jena, Germany; Apomixis Research Group; Department of Cytogenetics and Genome Analysis. Leibniz Institute of Plant Genetics and Crop Plant Research (IPK), Corrensstraße 3, 06466 Gatersleben, Germany

**Keywords:** Hypericum perforatum, Flower, Reproductive organs, Apomixis, Apospory

## Abstract

**Background:**

St. John’s wort (*Hypericum perforatum* L.) is a medicinal plant that produces important metabolites with antidepressant and anticancer activities. Recently gained biological information has shown that this species is also an attractive model system for the study of a naturally occurring form of asexual reproduction called apomixis, which allows cloning plants through seeds. In aposporic gametogenesis, one or multiple somatic cells belonging to the ovule nucellus change their fate by dividing mitotically and developing functionally unreduced embryo sacs by mimicking sexual gametogenesis. Although the introduction of apomixis into agronomically important crops could have revolutionary implications for plant breeding, the genetic control of this mechanism of seed formation is still not well understood for most of the model species investigated so far. We used Roche 454 technology to sequence the entire *H. perforatum* flower transcriptome of whole flower buds and single flower verticils collected from obligately sexual and unrelated highly or facultatively apomictic genotypes, which enabled us to identify RNAs that are likely exclusive to flower organs (*i.e*., sepals, petals, stamens and carpels) or reproductive strategies (*i.e*., sexual *vs*. apomictic).

**Results:**

Here we sequenced and annotated the flower transcriptome of *H. perforatum* with particular reference to reproductive organs and processes. In particular, in our study we characterized approximately 37,000 transcripts found expressed in male and/or female reproductive organs, including tissues or cells of sexual and apomictic flower buds. Ontological annotation was applied to identify major biological processes and molecular functions involved in flower development and plant reproduction. Starting from this dataset, we were able to recover and annotate a large number of transcripts related to meiosis, gametophyte/gamete formation, and embryogenesis, as well as genes that are exclusively or preferentially expressed in sexual or apomictic libraries. Real-Time RT-qPCR assays on pistils and anthers collected at different developmental stages from accessions showing alternative modes of reproduction were used to identify potential genes that are related to plant reproduction *sensu lato* in *H. perforatum*.

**Conclusions:**

Our approach of sequencing flowers from two fully obligate sexual genotypes and two unrelated highly apomictic genotypes, in addition to different flower parts dissected from a facultatively apomictic accession, enabled us to analyze the complexity of the flower transcriptome according to its main reproductive organs as well as for alternative reproductive behaviors. Both annotation and expression data provided original results supporting the hypothesis that apomixis in *H. perforatum* relies upon spatial or temporal mis-expression of genes acting during female sexual reproduction. The present analyses aim to pave the way toward a better understanding of the molecular basis of flower development and plant reproduction, by identifying genes or RNAs that may differentiate or regulate the sexual and apomictic reproductive pathways in *H. perforatum*.

**Electronic supplementary material:**

The online version of this article (doi:10.1186/s12864-015-1439-y) contains supplementary material, which is available to authorized users.

## Background

Reproduction is the highest priority of all living things, and involves a complex combination of processes whose variability between species has long puzzled evolutionary biologists. In angiosperms, reproduction for the most part culminates in the formation of male and female gametophytes, whose union leads to a new sporophyte generation that will begin from seeds. In the flower, male and female sporogenesis and gametogenesis generate two types of gametes that are either enclosed in an embryo sac (egg cell), or pollen grain (sperm).

Despite the enormous complexity and heterogeneity of flower morphology existing within angiosperms, a number of recent publications focusing on the genes involved in flower development suggested that the biological basis of flower development is actually highly conserved among plants [1]. Accordingly, the genetic and epigenetic bases of gamete formation have been studied in model species, such as *Arabidopsis*, snapdragon and maize, using a number of different approaches. The production and characterization of mutants defective for their ability to set seeds [2,3], in addition to expression studies [4], led to the identification of several genes that are potentially or effectively associated with sporogenesis and gametogenesis [5]. A number of mutations affecting the normal progression of meiosis [6] and gametophyte development [2,7], as well as double fertilization and seed development [8], have been studied in great detail (reviewed by [5]). Interconnections between the ovule and the developing embryo sac have emerged in a number of studies in which sporophytic mutations directly affecting gametophytic development were studied [9-11]. More recently, the mutation of genes involved in the production and action of sRNAs in the ovule have been shown to have dramatic effects on the development of the embryo sac [12,13], thus supporting the idea that sRNA-related mechanisms residing in the sporophyte are critical for cell-fate determination in the ovule and the formation of gametes [13-15].

Increasing interest has been focused on *Hypericum perforatum* for the study of apomixis, a naturally occurring form of asexual reproduction whereby progeny inherit the entire maternal genome through the seed [16-20]. *Hypericum* spp. (1.3 pg/2C, equal to ∼630 Mb) have *x* = 8 chromosomes: individuals are mainly tetraploid with 2*n* = 32, but diploid and hexaploid individuals have been found. *H. perforatum* is an invasive perennial herb that is widely distributed in a variety of habitats and is regarded as a serious weed in many countries [21,22]. Several compounds produced by *Hypericum* species have stimulated the interest of the scientific community for their biological activity [23], and *H. perforatum* has been studied for the identification of potential genes involved in the biosynthesis of active metabolites [24]. From the reproductive point of view, *H. perforatum* reproduces via aposporic apomixis, a gametophytic variant (according to Nogler, [25]) whereby the alternative differentiation of a somatic cell gives rise to a functional, unreduced embryo sac. In principle, aposporic initial cells in apomictic plants are somatic cells belonging to the nucellus, which change their fate by being able to mitotically divide and develop functional embryo sacs through mimicking sexual gametogenesis development [19,26]. The mode of reproduction in *H. perforatum* is highly dynamic, and biotypes span from almost complete sexuality to nearly obligate apomixis. In particular, apomixis is mostly found in tetraploid individuals and is characterized by complete penetrance and variable levels of expressivity, ranging from 20% to 97% (for review, see Pupilli and Barcaccia, [27] and ref. therein). The occurrence of diploid and hexaploid individuals reflects a dynamic reproductive system because haploidization and polyploidization are mediated by parthenogenesis of meiotic egg cells and fertilization of aposporic egg cells, respectively [17,26]. As with other asexual plant complexes, apomixis and hybridization are closely linked in *H. perforatum* [28], and interestingly the dosage of genetic factors has been proposed to influence the penetrance of apomixis, as tetraploid and hexaploid genotypes tend to be more apomictic and sexual respectively, regardless of geographic origin [29]. These observations are in agreement with the hypothesis that apomixis might relies upon spatial or temporal mis-expression of genes acting during female sexual reproduction [30].

*Hypericum perforatum* is considered an attractive model system for the study of apomixis because it is characterized by a relatively small genome size, the availability of morphologically distinct ecotypes, self-compatibility and easy cross-ability, high degree of molecular polymorphisms, along with a versatile mode of reproduction, a relatively short generation time and an abundant seed set [26,31]. Genotypes that produce embryos either from aposporic fertilized egg cells or from parthenogenesis of meiotically reduced egg cells have been identified, suggesting that apospory and parthenogenesis may be developmentally uncoupled [17]. It is now well known that parthenogenic capacity is preferentially expressed by aposporic egg cells (or, alternatively, non-parthenogenic behavior is frequently associated with meiotic egg cells). Nevertheless, aposporic egg cells can frequently occur in non-parthenogenic individuals, and parthenogenic development of meiotic egg cells can also take place. Genotypes that almost exclusively express only one component of apomixis while suppressing the other support the hypothesis that two distinct genetic factors control apospory and parthenogenesis in this species (for review, see Barcaccia *et al*., [27] and ref. therein). Recently, the genetic basis of apomixis in *H. perforatum* was uncovered by mapping and then sequencing a locus (designated *HAPPY* for *Hypericum APOSPORY*) associated with apospory, demonstrating that distinct genetic factors are associated apospory and parthenogenesis in this species [18].

Recently, next generation sequencing (NGS) technologies have been used to investigate gene expression changes associated with sperm development [32], differentiation of the megaspore mother cell [33] and specific cell types of the embryo sac [34] in *Arabidopsis*. In an alternative approach, high-quality single nucleotide polymorphisms (SNPs) were mined from NGS libraries sequenced from a number of sexual and apomictic *Ranunculus* genotypes in order to elucidate the origin and evolution of apomixis [35]. Taken together, the availability of reference DNA and RNA datasets are increasing the ease at which complex phenotypes and processes in non-model organisms can be analyzed. Here we sequence and annotate the flower transcriptome of *H. perforatum* with particular reference to processes related to reproduction. The annotation and comparative investigation of the flower transcriptome is a critical step for better understanding the genetic control of apomixis, and provides valuable information on gametophyte determination and gamete formation in *Hypericum*.

## Results

### *De novo* assembly and annotation of the *Hypericum* flower transcriptome

**Figure 1:**
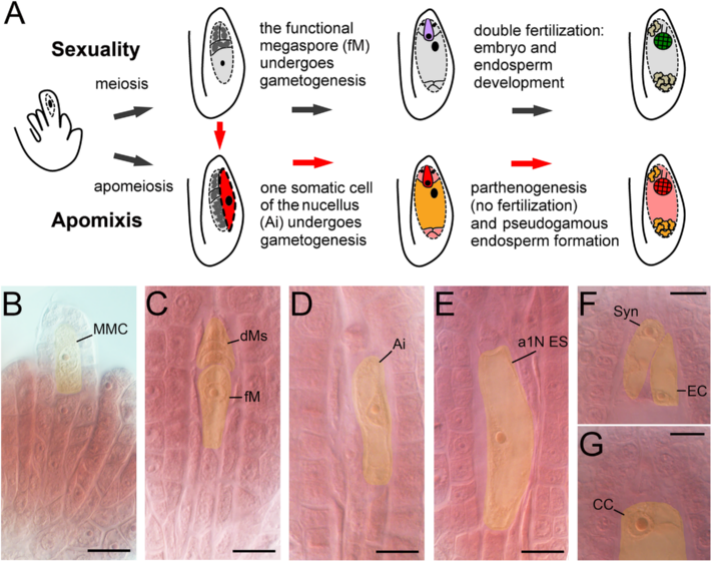
**Developmental stages of** ***H. perforatum*** **ovules sampled and analyzed in this research. A**: Schematic representation of key aspects of sexual and apomictic reproductive pathways in *H. perforatum* and key developmental stages. **B**-**G**: Ovules at developmental stages spanning meiosis, aposporic initial formation and development, and gametogenesis. **B**: megaspore mother cell (MMC); **C**: tetrad showing a functional megaspore (fM) along with three degenerating megaspores (dM); **D**: aposporic initial (Ai) cell; E: enlarged aposporic cell equivalent to an aposporic one-nucleate embryo sac (a1N ES); **F**: oangic apparatus showing in focus one synergid (Syn) and the egg cell (EC); **G**: detail of a central cell (CC) from a mature embryo sac. In each photograph the yellow area mark a given germinal cell type (each bar is 8 μm).

We analyzed two sexual and three apomictic accessions (two obligate and one unrelated facultative apomictic accession characterized by 24% apomictic seed development; Table 1; Figure 1, panel A). Flower buds and flower organs were collected at different developmental stages corresponding to *Arabidopsis* flower stages 1-12 and spanning meiosis and gametogenesis processes (Figure 1, panels B-G, with particular reference to the female gametophyte). Samples were collected to include the entire developmental pathway of gamete production, whose critical steps in sexual ovules include megaspore mother cell differentiation (Figure 1, panel B), its meiotic division to form a functional megaspore (Figure 1, panel C) and mitotic divisions giving rise to a functional eight-nucleate embryo sac with an egg cell apparatus (Figure 1, F-G). Conversely, aposporic ovules (Figure 1, panel I) are frequently characterized by a failure of the meiotic program and differentiation of one or multiple unreduced embryo sacs from the somatic cells (aposporic initials) of the ovule (Figure 1, panels D-E).

**Table 1:** **Information on the** ***H. perforatum*** **accessions used for flower transcriptome analyses**

**Accession**	**Description**	**Genealogy**	**Origin**	**Ploidy**	**Apospory**	**Reproduction**
13EU*°	Experimental population	4(F12 X An)1/4	IPK-Gatersleben (D)	2n = 4×	<4%	Sexual
20EU°	Experimental population	4(F12 X An)1/6	IPK-Gatersleben (D)	2n = 4×	<4%	Sexual
36EU*	Experimental population	4(F12 X An)1/9	IPK-Gatersleben (D)	2n = 4×	<4%	Sexual
141EU°	Experimental population	4(F12 X No)1a/39	IPK-Gatersleben (D)	2n = 4×	<4%	Sexual
Hp4/13*	Wild population	n.a.	Feltre (I)	2n = 4×	24%	Facultative apomictic
39EU*	Experimental population	4(F12 X No)1a/1	IPK-Gatersleben (D)	2n = 4×	>96%	Obligate apomictic
222EU°	Experimental population	4(R1C2 X Si)1c/2	IPK-Gatersleben (D)	2n = 4x	>96%	Obligate apomictic
1886US°	Wild population	n.a.	Iron Mountain (USA)	2n = 4x	>96%	Obligate apomictic
1973US*	Wild population	n.a.	Tecumseh (USA)	2n = 4×	>96%	Obligate apomictic
3348EU°	Wild population	n.a.	Hamburg (D)	2n = 4×	>96%	Obligate apomictic

For each plant accession, the origin, ploidy and degree of apomixis are indicated. Accessions marked with *were used for 454 sequencing, while those with °were used for Real-Time RT-qPCR. Apospory expressed as percentage was determined by flow cytometric screening of 48 single seeds. For details on the origin and composition of experimental populations please refer to [18].

All full-length enriched cDNA libraries were successfully normalized (data not shown) in order to avoid over representation of the most commonly transcribed genes and to maximize sequence diversity. Roche 454 sequencing of two full plates yielded a total of 1.47 million sequences, and an average number of sequenced reads from each library of 177,822 (Additional file 1: Table S1).

High quality sequences from all libraries were pooled and assembled to create a single global reference transcriptome, and yielded 33,860 isotigs with an average length of 1,002 bp and an N50 of 1,184 bp (Table 2). Following the assembly procedure, the quote of singletons was equal to 2% (Table 2). As a result, the assembly pipeline produced 60,428 assembled sequences - from now onward defined as unigenes - that were composed of 33,860 isotigs (accounting for 98% of sequenced reads) and 26,568 singletons (Table 2).

**Table 2:** **Descriptive statistics related to the reference assembly**

**Assembled reads**	**1,248,201 (87.7%)**
Number of Isotigs	33,860
Singletons	26,568 (1.9%)
Repeat/Outlier/Tooshort	76,514 (5.6%)
Average size of Isotigs	1,002
N50 Isotig size	1,184
Larger Isotig	1,4574
Average number of Reads/Isotigs	40.20

The reference assembly was built starting from the pool of reads sequenced from each library.

BLASTX analysis aligned approximately 61% of the *Hypericum* unigenes to a protein present in the non-redundant (nr) database (n = 36,988; E-value ≤1.E-06; Table 3). The remaining 39% of assembled unigenes (n = 23,455; Table 3) did not yield significant matches at this level of stringency. The relative contribution of assembled and non-assembled reads to the annotation of the *Hypericum* flower unigenes was also computed with a BLASTX-based procedure (see Additional file 1: Table S2).

**Table 3:** **Number of hits resulting from BLASTX analyses of the assembled** ***Hypericum*** **sequences**

**Species**	**Hits**	**BLAST (%)**
*Ricinus communis*	12,950	35.0%
*Populus trichocarpa*	11,887	32.1%
*Vitis vinifera*	4,329	11.7%
*Glycine max*	1,788	4.8%
*Medicago truncatula*	734	2.0%
*Arabidopsis thaliana*	660	1.8%
*Arabidopsis lyrata*	384	1.0%
*Lotus japonicas*	223	0.6%
*Jatropha curcas*	186	0.5%
*Hypericum perforatum*	183	0.5%
Others (632 Taxonomic IDs)	3,664	9.9%
Sequences with Blast Results (*)	36,988	61.2%
Sequences without Blast Results (*)	23,455	38.8%

BLAST (%) is referred to the total number of sequences having at least one BLAST hit, with reference to the whole set of assembled sequences.

As shown in Table 3, 90% of the best hits produced by BLASTX were concentrated in 10 species, whereas the remaining 10% of BLASTX hits were distributed over 632 taxonomic entities. Roughly 86% of the most significant alignments were made with proteins deduced from *Ricinus communis* (35%), *Populus trichocarpa* (32%), *Vitis vinifera* (12%), *Glycine max* (5%) and *Medicago truncatula* (2%; Table 3).

### Alignment of unigenes on the *HAPPY* locus and the use of this resource for the identification of allele/splice variants

**Figure 2:**
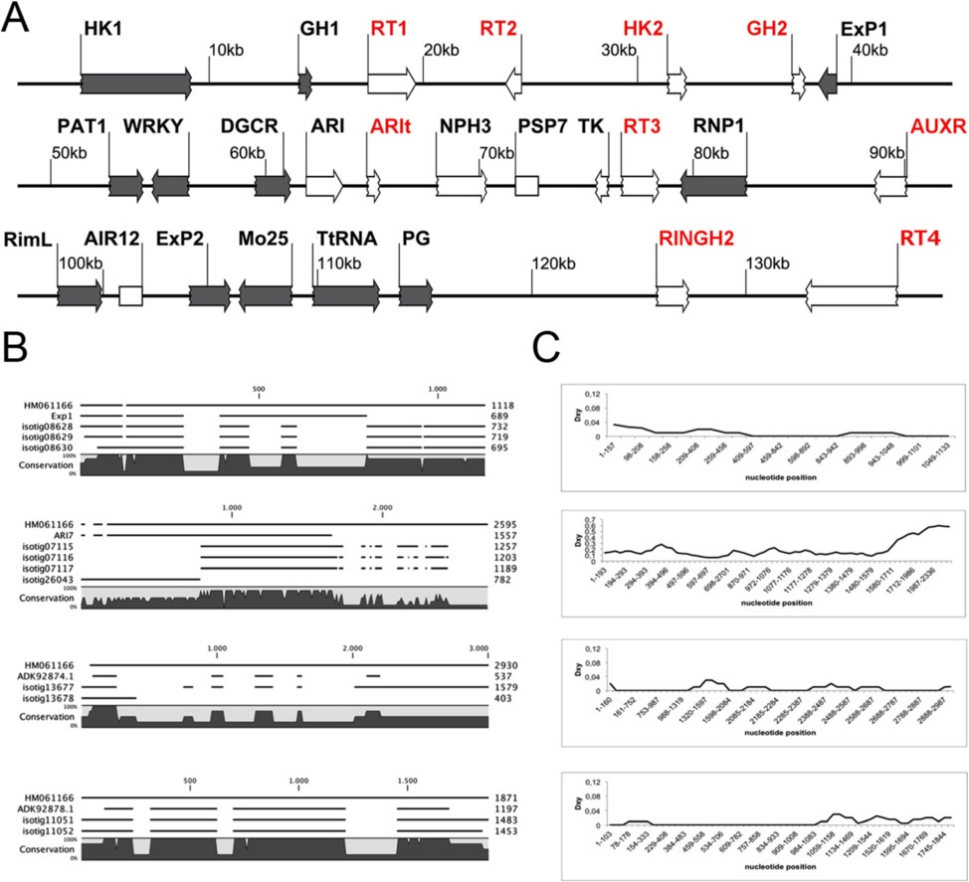
**Unigenes aligned to the BAC clone HM61166.1, containing the** ***HAPPY*** **(** ***Hypericum*** **apospory) locus** [18]**. A**: Gene composition of the BAC clone. The grey boxes indicate genes located in the chromosome 14 of poplar (*Populus trichocarpa*), while white boxes indicate genes located in other chromosomes. Gene names are indicated in black if aligned to one or multiple unigenes and in red if any unigenes aligned to the corresponding sequence. **B**: Alignments of multiple unigenes to the sequence extracted from HM61166.1 and the corresponding predicted CDS. **C**: Distribution of the average number of nucleotide substitutions per site (Dxy), calculated between unigenes and the aligned region of HM61166.1.

Screening for the identification of putative homologs in the nr database produced a number of matches with proteins encoded by genes located in the BAC clone HM061166.1 (see Additional file 1: Table S3) containing the genetic loci associated with apospory in *H. perforatum* (Figure 2, panel A). BLASTN searches of all 60,428 unigenes over the sequence of the BAC clone produced 17 relevant matches, 9 of which aligned to multiple unigenes (see Additional file 1: Table S3). Overall sequence identity shared by unigenes and the corresponding regions of HM061166.1 was on average 95%. Moreover, BLASTN searches did not produce reliable matches for the retrotransposons located in the BAC clone. The fact that the truncated genes ARI-T, HK2 and GH2 did not match none of the unigenes from our collection (Figure 2, panel A) may indicate that these genes were not expressed or were expressed at levels below our detection threshold. Alternatively, divergence between the genetic loci of the investigated plants (Figure 2, panel A) may have hampered the production of BLAST matches (see also [18]).

BLASTX matches from the nr protein database (Additional file 1: Table S3) also revealed possible synteny between the *Hypericum* sequence HM061166.1 and the chromosomal region located between the loci POPTR_0014s16260.1 and POPTR_0014s15970.1 of poplar (*Populus trichocarpa*). One exception to this was the region containing ARI and a number of other genes, which had truncated forms of ARI-T, NPH3 and TK that are not located in the same chromosome (LG14) in poplar (Additional file 1: Table S3; Figure 2). Positive matches of flower transcripts with nucleotide sequences of HM061166.1 further suggested the presence of some additional coding regions in the BAC clone, including PSP7 and AIR12.

Since distinct unigenes are the product of different assembly output, it is possible that the finding of multiple unigenes matching the same BAC portion reflects the existence of duplicated genes (*i.e*., paralogs) or allelic variation existing in our pool of genotypes. To test this hypothesis we selected 9 regions of the BAC clone HM061166.1 that shared high similarity with multiple unigenes (Figure 2; Table 4), aligned the sequences in multiple alignments and analyzed the nucleotide diversity existing between unigenes and reference sequence extracted from HM061166.1. As external controls, the same statistics were made for the three single copy regions ITS or internal transcribed spacers, matK and ndhF (Table 4). Nucleotide diversity displayed by unigenes when compared to their reference sequences extracted from HM061166.1 was an average 0.044 ± 0.062 (within unigenes: 0.025 ± 0.026). Conversely, the nucleotide diversity estimated for the control regions ITS, matK and ndhF was much lower and averaging 0.01 ± 0.01 (Table 4). The average nucleotide diversity measured in the BAC regions surrounding ARI7-like was as low as 0.013 ± 0.012 (within unigenes: 0.013 ± 0.016). Nucleotide diversity for sequences aligning to the ARI7-like gene was equal to 0.170 (within unigenes: 0.080). For a number of unigenes, the nucleotide diversity shared with genes encoding for WRKY-like protein, HnRNP, RimL/acetyltransferase-domain protein, PG was equal to 0.00 (Table 4). Despite the low genetic diversity, the unigenes aligned to the genes: Exp1, DGCR-like protein, HnRNP, RimL/acetyltransferase-domain protein, and Mo25-like protein displayed alignment variants featuring a possible lack or insertion of sequence traits in their CDS, coding sequences (Figure 2, panel B). The distribution of the average number of nucleotide substitutions per site (Dxy) calculated between unigenes and corresponding regions of HM061166.1 showed that nucleotide substitutions were concentrated in the 3’-end of the alignments in at least 50% of investigated regions (Figure 2, panel C, see also Additional file 2: Figure S1).

**Table 4:** **Diversity statistics for the unigenes aligning to the** ***Hypericum*** **BAC clone HM06166**

**Gene product**	**Accession**	**Unigenes**	**S/AL**	**Pi/AL**	**S/U**	**Pi/U**	**Hn/ALU**
Predicted protein	XP_002883670.1	isotig08628			7	0.007	4
		isotig08629					
		isotig08630					
WRKY-like protein	ADK92866.1	isotig10799	4	0.000	16	0.010	3
		isotig10800	11	0.010			
DGCR-like protein	ADK92867.1	isotig04455	9	0.010	21	0.022	3
		isotig04458	24	0.030			
ARI7-like	XP_002310443.1	isotig26043	98	0.160	139	0.081	4
		isotig07115	126	0.170			
		isotig07116	121	0.160			
		isotig07117	119	0.150			
Thymidine kinase (TK)	ADK92870.1	isotig09624	7	0.010	7	0.008	3
		isotig09625	7	0.010			
HnRNP	ADK92872.1	isotig31453	2	0.020	26	0.037	3
		isotig11295	3	0.000			
		isotig11296	20	0.030			
RimL/acetyltransferase-domain protein	ADK92874.1	isotig13677	4	0.010	0	0.000	2
		isotig13678	0	0.000			
Mo25-like protein	ADK92876.2	isotig05993	34	0.030	43	0.031	3
		isotig05994	36	0.030			
Polygalacturonase (PG)	ADK92878.1	isotig11051	3	0.000	17	0.012	3
		isotig11052	11	0.010			
ITS (nuclear)**		contig00041	1	0.002	n.a.	n.a.	2

Statistics are based on multiple unigenes aligning to unique coding sequences predicted from the BAC clone HM061166, containing the *HAPPY* locus associated to Apospory (AL). For each coding region (Gene product), the Unigene Id, the number of polymorphic sites (S/AL) and measured nucleotide diversity (Pi/AL) are reported. The number of polymorphic sites (S/U) and nucleotide diversity (Pi/U) calculated among unigenes are also indicated. The number of haplotypes emerging from the alignment of the multiple unigenes to coding sequences predicted from the BAC clone HM061166 is indicated as Hn/ALU. **Locus not included in the BAC clone HM061166, which was used as external reference for statistics on sequence genetic diversity. Unigenes were deposited as TSA project under the accession GBXG00000000.

### The distribution of sequences between single libraries

**Figure 3:**
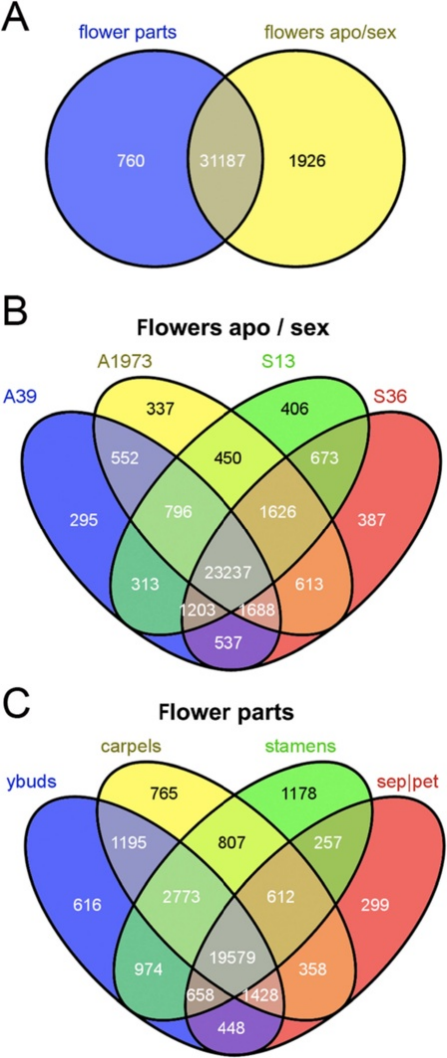
**Statistics on the composition of libraries and distribution of unigenes.** Venn diagrams that graphically represent the number of *H. perforatum* unigenes whose presence was shared among libraries, as indicated by the contribution of each library to the assembly. All possible intersections between libraries, as well as library specific sequences, are shown. **A**: Sequences shared by the two sequencing approaches; **B**: Sequences derived from whole flowers of two apomictic (A39, A1973) and two sexual (S13, S36) individuals; **C**: Sequences derived from single flower parts collected from a facultative apomictic accession. sep|pet: sepals and petals.

Of the 60,428 unigenes composing our reference assembly, and identified from different flowers and flower parts, 33,860 sequences were assembled with reads derived from one or multiple libraries. Calculation of the number of raw reads assembled in each unigene from each library allowed us to estimate the presence of each unigene in each sequenced library. These data were used to infer the overlaps existing between the two sequencing reactions (sequencing plates) and independent libraries (*i.e*., 4 libraries for each sequencing plate). About 92% of the assembled unigenes were composed of sequences shared between the two independent sequencing experiments (Figure 3, panel A). As much as 86% of unigenes were identified from the flowers of at least three sexual or apomictic individuals (Figure 3, panel B). Similarly, 78% of unigenes were shared by at least three libraries made from the different flower parts. Taken together these results indicate that most transcripts were detectable at the flower-transcriptome level, regardless of reproductive mode or specific flower organ, while numbers of transcripts detectable in single libraries were low (Figure 3, panels B-C).

The existence and abundance of overlaps between the libraries derived from flowers collected from sexual and obligate apomictic accessions were also measured (Figure 3, panel B). A total of 552 (1.7% of assembled unigenes) transcripts were present in both apomictic libraries, but were not found in the sexual libraries. Moreover, a total of 673 sequences (2.0% of assembled unigenes) were observed in the two sexual samples, but not in the apomictic samples.

When we considered the different flower parts collected from a facultative apomictic accession (characterized by 24% apospory), and hence showing either apomictic or sexual reproduction, we could identify 616 unigenes (1.9% of total unigenes) exclusive to young flower buds at stages preceding micro- and mega-sporogenesis (Figure 3, panel C). Moreover, 765 and 1,178 sequences were sequenced only in carpels and stamens, respectively (equal to 2.4% and 3.7% of total unigenes, respectively) (Figure 3, panel C). The overlap between carpels and stamens consisted of 807 (2.5%) single unigenes. Similarly, 1,195 and 974 transcripts were found in the overlapping regions of young buds with carpels and stamens, respectively.

Finally, most genes residing on the genomic region comprised in the BAC clone HM061166.1, thus surrounding the *HAPPY* locus, were expressed in the different flower parts as well as in both sexual and apomictic flowers analyzed in this study, as indicated by the production of multiple sequence reads matching the CDS predicted from the genomic sequence HM061166.1 (see Additional file 1: Table S4).

### Ontological annotation of sexual and apomictic specific sequences

**Figure 4:**
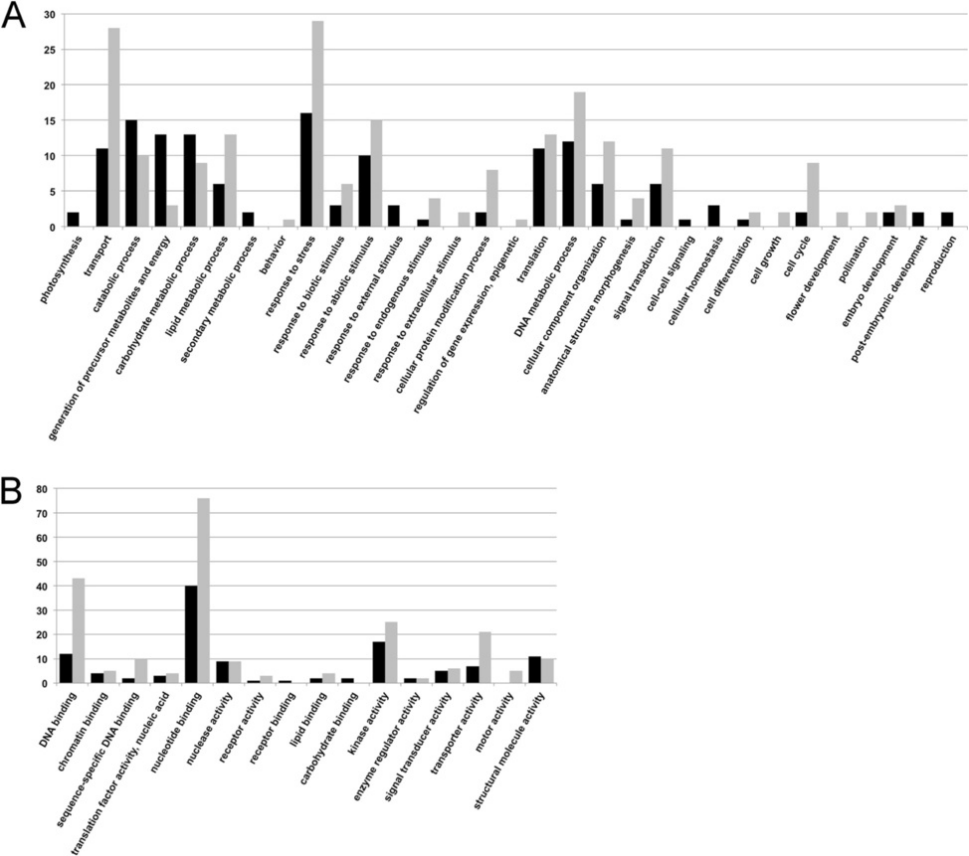
**Annotation of sequences detected only in apomictic and sexual libraries.** Annotation of apomictic- and sexual-specific sequences according to the GO vocabularies. **A**: GO terms associated with biological processes; **B**: GO terms associated with molecular functions. Overall annotations refer to 336 sexual-specific and 204 apomictic-specific unigenes that could be annotated with single or multiple ontological terms. The number of annotated sequences is shown as black bars for the apomictic libraries and gray bars for the sexual libraries.

For the annotation of gene products with a possible key role in plant reproduction and seed formation, we checked the ontological annotation associated to all unigenes identified only in sexual and apomictic libraries (673 and 552 unigenes, respectively; Figure 3). Metrics concerning the full ontological annotation of the flower transcriptome are reported on Additional file 1: Table S5. Most annotations associated to unigenes identified only in sexual and apomictic libraries were shared between the two sequence collections, although some extent of variability was observed for single GO annotations (Figure 4).

Highest scores were recorded for terms associated to the sensing and response stresses and stimuli as well as terms associated to metabolism of carbohydrate, lipids and secondary compounds (Figure 4, panel A). The annotations: cell differentiation (hits: 3, GO:0030154), cell cycle (hits: 11, GO:0007049) and embryo development (hits: 5, GO:0030154) were also identified at lower extend. Few sequences identified only in the sexual libraries could be annotated as regulation of gene expression, epigenetic (hits: 1, GO:0040029), cell growth (hits: 2, GO:0016049), flower development (hits: 2, GO:0009908) and pollination (hits: 2, GO:). Likewise, cell-cell signaling (hits: 1, GO:0007267), cellular homeostasis (hits: 3, GO:0019725), post-embryonic development (hits: 2, GO:0009791) and reproduction (hits: 2, GO:0000003) were associated to a small number of sequences specific of the apomictic libraries (Figure 4, panel A). As far as the molecular function of unigenes identified only in sexual and apomictic libraries is concerned, the most frequent annotations were DNA binding (hits: 55, GO:0003677), nucleotide binding (hits: 116, GO:0000166), kinase activity (hits: 42, GO:0016301) and transporter activity (hits: 28, GO:0005215) (Figure 4, panel B).

### Identification of genes involved in flower development, gamete formation and plant reproduction

As flower organs were sampled in this study, we expected the libraries to contain genes involved in flower development, gamete formation and reproduction. To test this hypothesis and annotate the unigenes, we created a database of 811 *Arabidopsis* genes whose function has been associated with plant reproduction *sensu lato* and used this set of data as a source of data specifically focused on reproduction (see Additional file 1: Table S6). BLAST analyses indicated that 1,674 *Hypericum* unigenes were putative orthologs to 632 *Arabidopsis* gene products (78% of total considered genes associated to reproduction in the broad sense).

**Figure 5:**
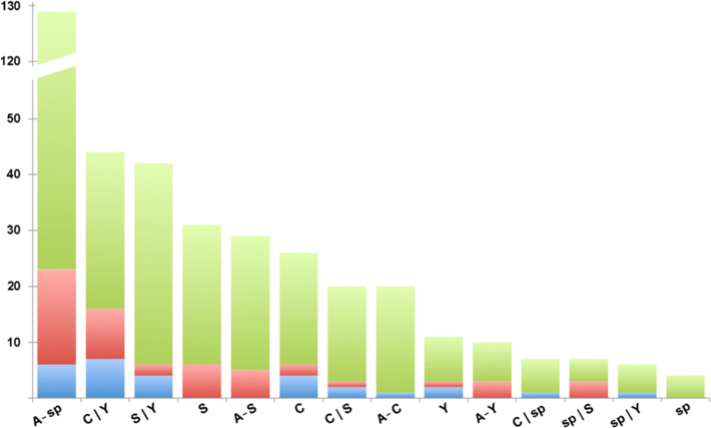
**Abundance of sequences detected only in apomictic or sexual libraries.** Distribution of reproduction-related unigenes according to the different flower parts and reproductive behaviors of *H. perforatum*. Legend: red, sequenced only in sexual libraries; blue, sequenced only in apomictic libraries; green, sequenced from both sexual and apomictic libraries. C: Carpels; S: Stamens; sp: Sepals/Petals; Y: Young buds; A-: present in all verticils but the one not assessed. Sequences shared by two libraries are indicated with the name of the libraries separated by “|”.

An attempt to join the annotation of reproductive-related genes to their expression site was done by checking the distribution of sequences matching reproduction-related genes within the different flower parts. Approximately 63% of the sequences matching reproduction-related genes were shared among all organs. Of the remaining sequences, most were expressed in all flower tissues, with the exception of only the sepals and petals (Figure 5). To a lesser extent, sequences were present in carpels and young buds and stamens and young buds, followed by stamens and carpels alone (Figure 5). According to this finding, the distribution of apomictic- or sexual-specific unigenes in each plant organ revealed that nearly half of the annotated unigenes belonged to three groups: i) young buds, carpels and stamens; ii) carpels and young buds; and iii) stamens and young buds. Approximately 10% of the apomictic- or sexual-specific unigenes could not be paired with any specific flower part (Figure 5).

Among the sequences annotated as reproduction-related, we could effectively annotate numerous unigenes involved in sporogenesis, gametogenesis and embryogenesis (Table 5). NCBI and TAIR searches yielded 89% of the annotations related to meiosis, gametogenesis and embryogenesis (Table 5). Sequences derived exclusively from sexual libraries produced almost double the number of reproductive related genes derived from the apomictic libraries (223 and 118 unigenes, respectively). This difference was particularly evident for genes comprised in the two categories: “regulation of megasporogenesis” (17 and 5 unigenes, respectively) and “female gametogenesis, fertilization and seed development” (21 and 3 unigenes, respectively) (Table 5).

**Table 5:** ***Hypericum*** **matches to the plant reproductive database of** ***Arabidopsis***

**Unigenes**	**At loci**	**Biological terms**	**APO**		**SEX**		**Common**
			**1 repl.**	**2 repl.**	**1 repl.**	**2 repl.**	
20	9	Cell specification	0	1	1	2	16
90	36	Regulation of megasporogenesis	3	2	16	1	68
9	4	Regulation of megagametogenesis	1	0	0	0	8
79	24	Female gametogenesis, fertilization, seed development	3	0	21	2	53
21	1	Pollen tube reception	0	0	3	0	18
26	10	Seed development and size regulation	5	1	3	0	17
9	3	Imprinted and imprinting related genes	0	0	0	2	7
54	16	Sporophytic mutants with gametophytic defects	1	2	3	3	45
7	2	Gametophytic factor	0	0	2	1	4
25	10	Unfertilized embryo sac	2	1	1	0	21
93	36	Maternal effect embryo arrest	5	1	10	2	75
482	164	Embryo defective	30	2	43	8	399
81	29	Meiosis	8	1	15	6	51
521	183	Gametogenesis	37	3	50	5	426
157	49	Embryogenesis	6	3	19	4	125
1,674	576	Total	101	17	187	36	1,333

The reproductive database was built upon TAIR and NCBI annotations and provides information on genes involved on reproductive processes. For each biological term or process, the number of *Hypericum* unigenes having a significant match with entries on the reproductive database is shown (unigenes). The number of reference *Arabidopsis* genes matched by a *Hypericum* unigene are also reported (At loci). For each biological term or process, the distribution of the *Hypericum* unigenes among the apomictic and sexual 454 libraries are shown. APO: present only in apomictic libraries; SEX: present only in sexual libraries; Common: present in both apomictic and sexual libraries. 1 repl: detected from a single library; 2 repl: detected in either apomictic (APO) or sexual (SEX) libraries.

With this approach, we were able to identify and annotate *Hypericum* unigenes whose expression has been associated with phases of ovule development (*e.g., INO*, *BEL*, *ANT*), along with transcripts whose activity is restricted or specific to cell fate in the ovule, such as in the nucellar tissue (*e.g*., *AGO9*, *RDR6* and *SGS3*) and embryo sac (*e.g*., *ATO*, *CLO*, *BLH1* and *LIS*) (Table 6). Similarly, we could associate 81 unigenes expressed in the *Hypericum* flower to meiosis (see Table 5). Among those, we detected and annotated a number of genes with known functions in meiosis (*e.g*., *DMC1*, *ATREC8*, *MMD1* and *MS5*), megaspore selection and gamete formation (*e.g*., *AGP18*, *AGO9*, *AGO5* and *MYB98*) (Table 6). Genes involved in the control of DNA methylation in sporophytic and gametophytic plant tissues, such as *MET1* and *DME*, were also identified and annotated (Table 6). Similarly, 521 and 157 unigenes were linked to gametophyte and gamete development and embryogenesis, respectively (see Table 5). In these categories we could sequence and annotate a number of genes whose mutants are known to affect the embryo sac, embryo and endosperm development (*e.g*., *AGL32*, *IAA32*/*MEE10*, *dVPE*, *FUS2*, *SERK*, *TANMEI*/*EMBR2757* and *ILA*) (Table 6).

**Table 6:** **Unigenes involved in key biological steps of ovule, gamete and seed formation**

**Unigene**	**Gene**	**Biological process**	**DB source**	**Libraries of flower parts**				**Apomictic libraries**	**Sexual libraries**
				**C**	**S/P**	**St**	**YB**	**mean (st.dev.)**	**mean (st.dev.)**
isotig28792	HpANT	Sporophytic mutants with gametophytic defects and seed development and size regulation	2; 8	1.5	0.0	0.0	3.0	0.0 (0. 0)	0.9 (0.0)
isotig03676	HpBEL1	Sporophytic mutants with gametophytic defects	2	2.1	0.0	0.0	0.0	3.1 (0.4)	1.8 (0.2)
isotig24625	HpINO	Sporophytic mutants with gametophytic defects	2	10.5	0.0	0.0	0.0	4.4 (0.0)	2.1 (0.3)
isotig30848	HpAGO9	Cell specification and regulation of megasporogenesis	1; 3; 4	0.0	2.3	0.0	2.4	4.2 (3.4)	0.0 (0.0)
isotig08685	HpRBR	Cell specification and regulation of megagametogenesis	1; 3; 4; G; T	0.0	0.0	0.0	0.0	0.0 (0.0)	2.5 (0.3)
isotig21033	HpRDR6	Regulation of megasporogenesis	3; 4	2.5	1.4	1.1	5.5	3.9 (4.5)	2.1 (0.0)
isotig18046	HpSGS3	Regulation of megagametogenesis	3; 4	8.5	7.1	6.7	8.1	5.3 (0.7)	6.4 (5.5)
isotig27635	HpATREC8	Cell specification and plant meiotic genes	1; 6; T	0.5	0.0	3.1	3.9	0.0 (0.0)	1.9 (1.5)
isotig06846	HpDMC1.1	Plant meiotic genes	6; T	1.0	0.0	2.6	3.4	1.7 (1.3)	3.5 (1.0)
isotig06845	HpDMC1.2	Plant meiotic genes	6; T	1.8	0.0	3.0	4.1	1.9 (1.7)	4.1 (1.5)
isotig30894	HpMND1	Plant meiotic genes	6; G; T	3.1	0.0	0.0	1.2	6.9 (6.4)	1.1 (0.1)
isotig31045	HpMS5	Plant meiotic genes	6; T	2.5	0.0	2.1	0.0	9.3 (10.6)	0.0 (0.0)
isotig32956	HpAGP18	Female gametogenesis, fertilization, seed development	5	2.5	0.0	0.0	0.0	8.1 (9.3)	1.4 (1.9)
isotig10679	HpAGO5.1	Regulation of megagametogenesis	7	3.7	0.6	6.1	2.0	4.6 (1.9)	6.6 (3.4)
isotig15447	HpAGO5.2	Regulation of megagametogenesis	7	5.4	13.0	6.6	8.2	4.4 (1.0)	4.6 (3.5)
isotig24484	HpMET1	Regulation of megagametogenesis and seed development	3; 4; 8	0.7	0.6	0.8	2.6	3.1 (1.7)	2.0 (1.1)
isotig09834	HpDME.1	Imprinted genes and imprinting related	3; 4	0.8	0.0	5.3	0.4	0.0 (0.0)	3.9 (2.7)
isotig09835	HpDME.2	Imprinted genes and imprinting related	3; 4	0.9	0.0	6.3	0.4	0.0 (0.0)	4.6 (3.3)
isotig31020	HpMYB98	Regulation of megagametogenesis, fertilization, seed development	3; 4; 5; T	0.0	5.8	0.0	0.0	0.6 (0.8)	0.6 (0.8)
isotig23549	HpDVPE	Female gametogenesis, fertilization, seed development	5	0.0	0.0	2.1	0.0	2.6 (0.5)	3.9 (1.2)
isotig33211	HpFUS2	Female gametogenesis, fertilization, seed development	5	0.0	0.0	1.0	0.0	0.0 (0.0)	6.1 (8.6)
isotig25989	HpILA	Female gametogenesis, fertilization, seed development	5	11.2	9.4	7.5	3.7	6.9 (1.2)	5.5 (6.8)
isotig23192	HpAGL32	Female gametogenesis, fertilization, seed development	5	14.3	0.0	0.0	0.0	7.9 (1.9)	6.4 (2.1)
isotig11347	HpSERK1	Gametogenesis	T	5.1	0.4	1.9	2.1	5.8 (1.0)	2.7 (0.2)
isotig11348	HpSERK2	Gametogenesis	T	2.9	2.5	1.8	5.0	5.0 (2.3)	3.2 (0.2)
isotig32841	HpIAA32	Maternal effect embryo arrest and gametogenesis	G; T	0.8	0.0	0.0	0.0	4.8 (0.3)	0.0 (0.0)
isotig05393	HpARF2	Seed development and size regulation	8	2.3	2.3	4.1	3.0	3.9 (0.4)	7.5 (0.0)
isotig29668	HpAGL62	Seed development and size regulation	8	0.0	0.0	0.6	0.5	2.2 (1.7)	1.0 (0.1)
isotig29604	HpTANMEI	Embryo defective phenotype and embryogenesis	G; T	2.7	0.0	0.0	0.5	1.4 (0.6)	0.0 (0.0)
isotig25721	HpAHP4	Seed development and size regulation	8	0.0	2.1	0.0	0.0	8.0 (1.3)	18.6 (13.1)

Genes were selected on the basis of their annotation. The number of reads, expressed as RPK (number of reads per kilobase of the reference CDS, normalized over the total number of sequences produced in the specified library and expressed as thousands of sequences), is displayed for each flower parts and for the two reproductive strategies (DB sources: 1, [12]; 2, [11]; 3 [5]; 4 [3]; 5, [4]; 6, [10]; 7, [13]; 8 [8]; G, GenBank; T, TAIR). Unigenes were deposited as TSA project under the accession GBXG00000000.

### Expression analysis of reproduction-related genes in sexual or apomictic plant accessions

**Figure 6:**
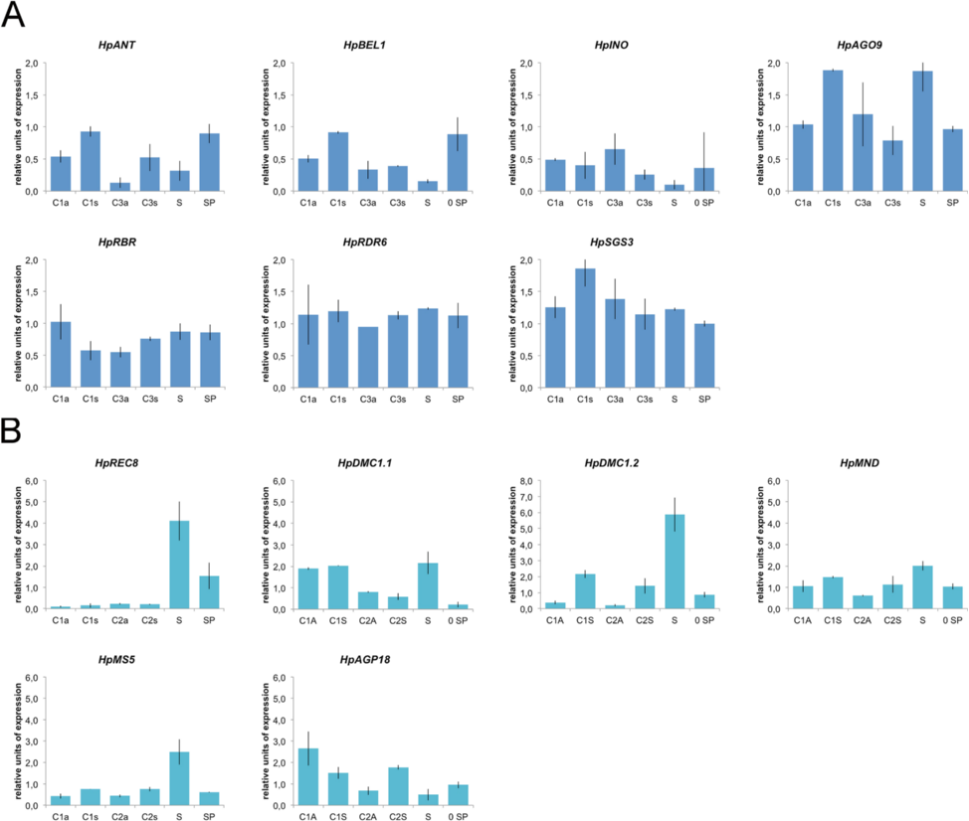
**Expression analysis of genes involved in meiosis, spore selection and ovule differentiation.** Quantitative Real-Time PCR results for a number of *H. perforatum* genes selected on the basis of their annotation and potentially involved in sporogenesis and spore selection **(panel A)** or ovule or cell differentiation **(panel B)**. Gene expression values are expressed in arbitrary units normalized against the level of expression detected in sepals and petals (sample: SP). C1a, C1s: flower stage 11; C2a, C2s: flower stage 12a, 12b; C3a, C3s: flower stage 12c-14. C1a, C2a, C3a: apomictic carpels; C1s, C2s, C3s: sexual carpels. S: stamens at flower stages 11-12. SP: sepals and petals at flower stages 11-12.

Real-Time RT-qPCR assays were used to verify whether apomictic plants are affected in the expression of genes that are expected to be involved in sexual reproduction-related processes (Table 6). In particular, the expression of 30 unigenes was tested in pistils and stamens collected from sexual (*i.e*., meiotic) and apomictic (*i.e*., apomeiotic) accessions (Table 6; Figure 6, panel A). A sample composed by sepals and petals was also included in the analyses, as external reference.

The expression levels of genes involved in determination of cell and ovule identity was tested in apomictic and sexual pistils in order to investigate differences between meiotic and aposporic gametophyte development. In particular, we studied the expression of key genes involved in the generation and activity of small RNAs and putatively involved in the determination of cell identity in pre-meiotic and meiotic ovules (Figure 6, panel A). The expression data of *HpRDR6* and *HpSGS3*, two genes involved in the siRNA formation in plants, did not show any differential pattern between apomictic and sexual pistils (Figure 6, panel A). Noteworthy, *HpAGO9*, which is implicated in the development and maintenance of MMC identity in the *Arabidopsis* ovule, was down-regulated at early stages and up-regulated at late stages of pistil development in apomictic genotypes compared to sexual ones (Figure 6, panel A). Some genes potentially involved in ovule and cell identity, like *HpINO*, did not reveal any differential expression between sexual and apomictic genotypes. Other genes potentially involved in cell identity, including *HpANT* and *HpBEL1,* were down-regulated at early and late stages of pistil development in apomictic genotypes (Figure 6, panel A). The expression data of *HpDMC1.2*, *HpMND* and *HpMS5* did show that these three genes are down-regulated in apomictic genotypes at early and late stages of pistil development (Figure 6, panel B). While *HpDMC1.2* was differentially expressed between sexual and apomictic pistils, the related *HpDMC1.1* was expressed at comparable levels in apomict and sexual pistils (Figure 6, panel B).

**Figure 7:**
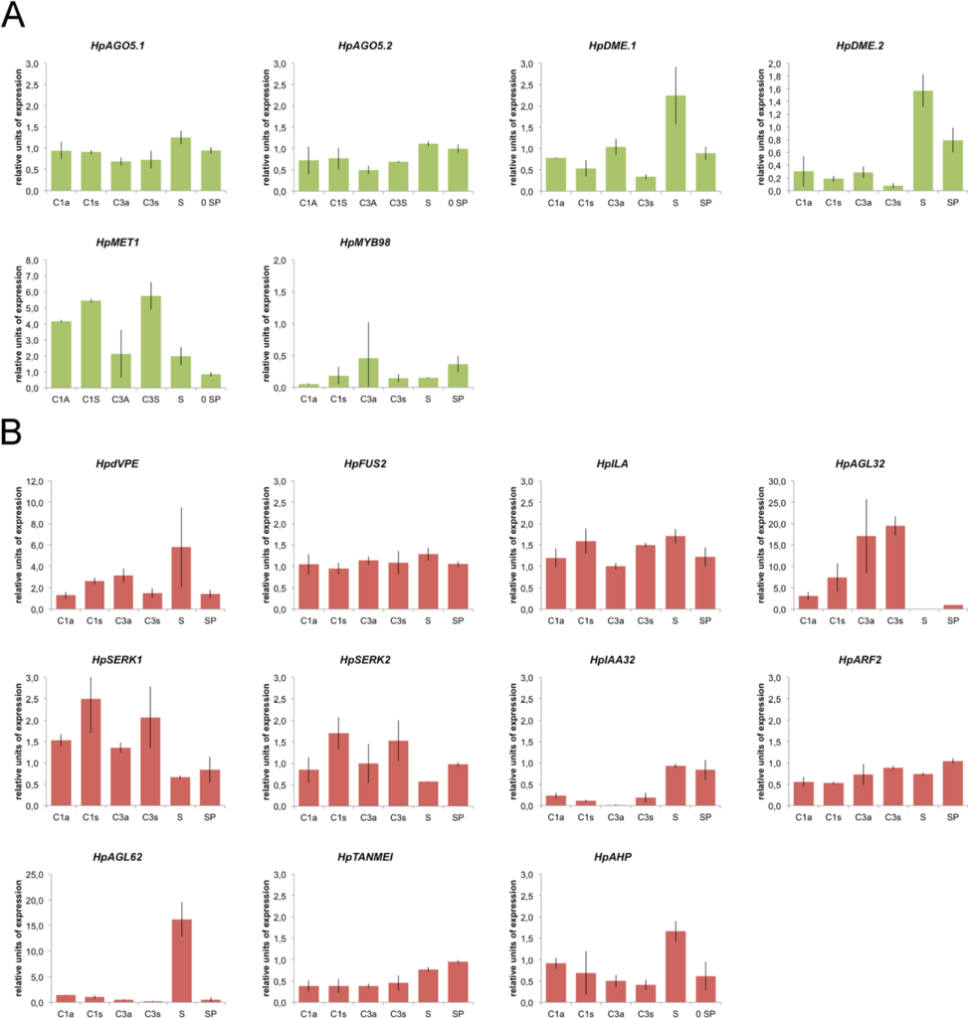
**Expression analysis of genes involved in gametogenesis, and embryo and embryo sac development.** Quantitative Real-Time PCR results for a number of genes selected on the basis of their annotation and potentially involved in gametogenesis **(panel A)** and embryo and endosperm development **(panel B)**. Gene expression values are expressed in arbitrary units normalized against the level of expression detected in sepals and petals (sample: SP). C1a, C1s: flower stage 11; C2a, C2s: flower stage 12a, 12b; C3a, C3s: flower stage 12c-14. C1a, C2a, C3a: apomictic carpels; C1s, C2s, C3s: sexual carpels. S: stamens at flower stages 11-12. SP: sepals and petals at flower stages 11-12.

Two *Hypericum ARGONAUTE* homologs of *AtAGO5*, both involved in megaspore selection and embryo sac development in *Arabidopsis*, showed no significant differences in expression levels between apomictic and sexual pistils (Figure 7, panel A). Concerning genes that in *Arabidopsis* control DNA methylation in sporophytic and gamethophytic tissues, *HpDME1* was found up-regulated in apomictic genotypes at early and late stages of pistil development, and to a lower extent also *HpDME2* was found up-regulated in apomictic genotypes (Figure 7, panel A). In addition, *HpMET1* proved to be down-regulated in apomictic genotypes compared to sexual ones at both stages of pistil development (Figure 7, panel A). The expression of several genes that are involved in embryo sac and embryo, and endosperm development (Table 6) was also tested in the same set of pistils from sexual and apomictic genotypes (Figure 7, panel B). It is worth mentioning that both *HpSERK1* and *HpSERK2* were differentially expressed in genotypes with antagonist reproductive features, being down-regulated at early and late stages of pistil development in apomictic genotypes (Figure 7, panel B). Moreover, with the only exception of *HpdVPE* and *HpILA*, whose expression in pistils appeared to differ in apomictic and sexual genotypes at late developmental stages, most of the tested genes did not reveal expression changes (Figure 7, panel B). The two genes *HpMYB98* and *HpAGL32*, known in *Arabidopsis* to be specifically expressed in ovule domains including the embryo sac and the endothelium, were found similarly expressed in apomictic and sexual pistils (Figure 7, panels A and B, respectively).

### Computational investigation of flower and seed-related genes based on *Arabidopsis* microarray-based reference transcriptome

The possibility to discover additional genes involved in molecular pathways leading flower and seed development was addressed by comparative approaches focused on *A. thaliana* flower and seed transcriptomes as references (see Additional file 1: Table S7). To do so we created an *Arabidopsis* microarray-based reference transcriptome, based on the expression data of 20 published microarray experiments performed in *A. thaliana* by using flower tissues at developmental stages preceding pollination (*e.g*., whole flowers, carpels and ovules, anthers and pollen grains). In addition to these, the microarray-based reference transcriptome was enriched with the data of 17 publically available microarray experiments performed on *A. thaliana* pollinated flowers and seeds (*e.g*., whole seeds, endosperm, embryo) (Additional file 1: Table S7).

Based on the published data of the *A. thaliana* microarrays, our reference dataset contained 20,372 genes expressed in at least one of the microarray experiments considered in our study, irrespectively of their expression values (Table 7). This reference dataset was used to set the architecture of pollinated and non-pollinated flowers in MapMan. On the other side, based on our BLASTX results, we could use approximately 60% and 63% of genes expressed in the *A. thaliana* flower and seed (12,035 and 12,003 hits, respectively) as they produced significant matches with one or multiple *Hypericum* unigenes (Table 7).

**Table 7:** **Coverage estimates of the** ***Hypericum*** **flower transcriptome by comparative analysis with** ***Arabidopsis*** **data**

**Plant organ (** ***Arabidopsis*** **)**	**Experiments (replicates)**	**Database size (# sequences)**	**Hits (%)**	**Hits with array elements that are not expressed in the specific experiments (%)**
Flower - not pollinated	21 (54)	20,032	11,970 (59.8)	331 (2.7)
Flower - pollinated	16 (40)	19,406	11,844 (61.0)	457 (3.7)
Flower - overall	37 (94)	20,372	12,035 (59.1)	266 (2.2)
Seed	50 (98)	20,679	12,003 (58.0)	298 (2.4)
Embryo	6 (12)	17,275	10,995 (63.6)	1,306 (10.6)
Endosperm	16 (34)	19,247	11,451 (59.5)	850 (6.9)

The transcriptome coverage was estimated by comparing annotated *Hypericum* sequences with publically available *Arabidopsis* microarray data (http://www.ebi.ac.uk/arrayexpress/). For each plant component, such as flower and seed, the number of microarray experiments and replicates that were used to estimate the size of each database are reported. The recovered hits indicate the number (and percentage) of database sequences that matched *Hypericum* accessions. For each plant component, the number of array elements that are not expressed in the specific experiments (non-significant hybridization signals), but that produced significant matches with *Hypericum* sequences is also reported.

We found that 191 *Hypericum* transcripts were expressed only before pollination in the *Arabidopsis* flower (see Additional file 3: Figure S2). Using the same approach, we could detect 65 *Hypericum* unigenes, which appeared to be expressed upon fertilization in *Arabidopsis* flowers (Additional file 3: Figure S2, panel A). Interestingly, a number of *Hypericum* unigenes were shown to be specific to certain seed structures in *Arabidopsis* (Additional file 3: Figure S2, panel B). This was the case of *HpAGL80*/*FEM111* (AT5G48670) and *HpEMB2271* (AT4G21130), whose *Arabidopsis* putative orthologs appeared to be expressed in the *Arabidopsis* endosperm.

An overview of metabolic and regulative pathways active during flower development was defined by mapping the *Hypericum* flower unigenes to the metabolic and regulative environments defined by MapMan (see Additional file 4: Figure S3). Particular emphasis was given to the maps: cell regulation overview and cellular response pathways (Additional file 4: Figure S3). A total of 74 apomictic-specific and 137 sexual-specific gene transcripts, whose expression was restricted to *Hypericum* flowers, had homologues in the *Arabidopsis* flower transcriptome (see Additional file 4: Figure S3, indicated as light-blue and green squares, respectively). Sixty-five *Hypericum* unigenes matched *Arabidopsis* seed-specific genes (see Additional file 4: Figure S3, red squares) whose annotations were related to the following categories: transcription factors (21 hits; 30%), protein modification (5 hits; 7%) and degradation (10 hits; 14%), receptor kinases (9 hits; 13%), as well as the synthesis and metabolism of hormones (13 hits; 19%; Additional file 4: Figure S3). Among the genes related to seed development in *Arabidopsis*, the homolog of the B3 family transcription factor AT1G26680 was uniquely present in the sexual libraries in *H. perforatum* (see Additional file 4: Figure S3, orange squares). Similarly, the expression of the homolog of the embryo-specific zinc-finger transcription factor AT5G0750 (*TZF6*), which is required for heart-stage embryo formation in the *Arabidopsis* seed, was present in the apomictic, but not in sexual carpels (see Additional file 4: Figure S3, violet squares).

## Discussion

### *De novo* sequencing and assembly of the *Hypericum* flower transcriptome

*H. perforatum* L. is a medicinal plant with reproductive behavior ranging from highly or nearly obligate apomixis (frequent) to complete sexuality (rare) in natural populations [19,31]. Even though *H. perforatum* is not a traditional model system for plant biology studies, it is a suitable system for the study of apomeiosis and parthenogenesis ([17-19,26]). To do so, we approached the flower transcriptome with two sequencing efforts conceived for better understanding how and where differential gene expression occurs between both reproductive forms. Hence, we sequenced cDNA collected from single flower organs (*i.e*., sepals and petals, stamens and carpels) at different stages of flower development, and linked these data to RNAseq data from whole flowers collected from two different obligately-sexual genotypes and two different highly-apomictic genotypes. These efforts led to the assembly of 1.47 million sequence reads to produce 60,428 unigenes, which proved to be a valuable tool for the study of the *Hypericum* flower transcriptome. Starting from these data, we were able to annotate and characterize 36,988 transcripts found expressed in male and/or female reproductive organs, including tissues or cells of sexual and apomictic flower buds. Our approach of sequencing flowers from two fully obligate sexual genotypes and two unrelated highly apomictic genotypes, in addition to different flower parts dissected from a facultatively-apomictic accession, enabled us to approach the complexity of the flower transcriptome according to its main reproductive organs as well as for alternative reproductive behaviors.

The assembly of such a large number of unigenes provided the possibility of testing for the presence of alternative alleles or splicing variants that could have been sequenced and assembled independently. To verify this thesis we investigated the unigenes aligning to the genomic region comprised in the BAC sequence HM061166.1 and surrounding the *HAPPY* locus [18]. Nine genes included in the BAC clone aligned to multiple unigenes sequenced from the flower libraries. Despite being sufficiently dissimilar to escape the assembly procedure, the fact that multiple unigenes aligned to the same region could indicate the sequencing of alternative alleles or splicing variants. This could be the case of the BAC regions encoding for the gene products *WRKY*, *DGCR* and *ARI7*, as well as *EXP1*, *RimL* and *PG*. Findings of nucleotide substitutions concentrated in the 3’-end of the alignments in at least 50% of investigated regions could be an additional clue that analyzed SNPs are robust and not attributable to sequencing artifacts.

On average, the nucleotide diversity of multiple unigenes mapping to the genomic window surrounding the *HAPPY* locus [18] was comparable to that estimated for the ITS regions used as external reference [36]. Despite the limitation due to the absence of mapping and quantitative expression data, the finding of nucleotide diversity shared by unigenes and genomic reference stretches comparable to the extent showed by ITS regions suggests that most of the unigenes may resemble allele variants rather than long-lasting duplicated genes (which would have been associated with higher nucleotide diversity, assuming enough time for independent accumulation of mutations). We are aware that coverage provided by the technology used in the study is lower than that recommended for robust variant detection analysis [35], and hence our estimates of nucleotide diversity could be affected by the presence of undetected of sequencing errors. Nevertheless, if we consider that the error rate is dependent on the sequencing method, its distribution across all loci of the BAC clone would be substantially uniform. Hence, we believe that our finding that unigenes aligning on ARI7-like displayed a nucleotide diversity largely exceeding that calculated on the surrounding regions of the BAC clone (0.013) is worth mentioning. This finding is interesting as we consider that ARI7 contains the actual marker co-segregating with apospory in *H. perforatum* and is among those few genes predicted from HM061166.1 (Additional file 1: Table S3).

Finally, taking into account the low genetic diversity existing within unigenes and CDS predicted from the BAC clone HM061166.1, the finding of alignment variants displaying the insertion or deletion of gene sequence traits in the unigenes provides a first indication for the presence of splice variants in sequences encoded by genes surrounding the *HAPPY* locus (Figure 2, see also Additional file 4: Figure S3). The allelic nature of these unigenes will be eventually confirmed by the availability of mapping data.

### Genes related to plant reproductive organs and seed formation

*De novo* sequencing of the *H. perforatum* flower transcriptome was attempted to identify genes related to plant reproduction, including transcripts specifically or preferentially expressed in anthers and/or pistils, and possibly differentially expressed between sexual and asexual meiosis and gametogenesis. Computational *in silico* annotation of flower transcripts provided us with an insight into the molecular mechanisms of various biological processes, including meiosis and gametogenesis.

Particular attention was paid to the annotation of genes important for cell specification [3,12] and genes whose mutants affect gametophyte development [5,8,11]. The orthologs of genes whose products are possibly involved in restricting cell-fate in ovules or gametophytes were detected (Table 6; Figure 6). Genes that are involved in regulation of the correct number of cell divisions occurring during megagametogenesis (*RBR*) [37] or the correct positioning of nuclei within the embryo sac (*BLH1*/*eostre*) [38] were also detected in our datasets. Accordingly, transcripts whose expression are thought to be crucial for selection of the functional megaspore and its cell identity (*AGP18*) [39], or those involved in proper specification of gametes and cell fates, including *LIS* [7], *CLO* [40] and *ATO* [40], were sequenced and annotated.

Overall, these findings demonstrated that our approach could successfully identify gene products whose expression is associated with ovule and seed development, and restricted to a few number of cells [41,42]. For example, we were able to identify the homolog of *MYB98* (Table 6; Figure 7), which in *Arabidopsis* is expected predominantly in the synergid cells and is considered to be critical for synergid differentiation in this species [41]. It is remarkable that *HpMYB98*, a gene associated with proper differentiation of synergid cells [3-5,17], was characterized by high levels of expression variation (compared to sexuals) in the pistils collected at late developmental stages from apomictic accessions (Table 6; Figure 7). As the sexuals and apomicts compared here were of identical ploidy, elevated gene expression variation in apomicts could be consistent with a number of factors including genetic background differences between genotypes [29], trans-acting regulatory variation in the hybrids or genomic differences having arisen due to sexuality versus apomixis [43]. Assuming that PCR efficiency was comparable in the different tissues, the high level of variation recorded in apomictic pistils at stages of late gametogenesis could be the result of spatial or temporal mis-expression of some components of the genetic pathways acting during gametogenesis.

The orthologs of genes that were previously associated with seed and endosperm abortion and anomalies in zygote development, including *IAA32/MEE10*, *dVPE*, *ILITHYIA*, *SERK1* and *SERK2* among others [4,44], were sequenced and studied in greater details by RT-qPCR. Quantitative expression studies demonstrated that apomictic and sexual pistils have different relative abundance of transcripts encoded by these genes (Table 6; Figure 7). The finding that multiple genes that are involved in seed and endosperm development are differentially expressed between sexual and apomictic pistils implies that entire reproductive gene pathways are modulated in their timing and levels during apomictic development.

Validation using RT-qPCR assays confirmed that *HpMET1* [45] and *HpDME* [46] are differentially expressed among the different flower parts (Figure 7) while only *HpMET1* appeared to be differentially expressed in apomictic and sexual pistils at developmental stages corresponding to gametogenesis. In *Arabidopsis MET1* absence leads to removal of the silencing methylation marks and the expression of genes (*e.g*., *FIS2* and *FWA*) involved in the regulation of endosperm development [5]. Accordingly, [45] reported that *MET1* is implicated in the repression of endosperm development in the absence of fertilization in *Arabidopsis* [45], while [47] provided data in support of the idea that a DNA methylation pathway in maize likely plays a critical role in the differentiation between apomictic and sexual reproduction. Hence, the reduction in the expression of *HpMET1* in apomictic pistils further support that variations of DNA methylation marks might be linked to gametophyte development in apomictic *H. perforatum*.

### Identification and expression analysis of candidate genes for apomixis

Apomixis, a form of asexual seed production is characterized by i) meiotically-unreduced egg cell formation (apomeiosis); ii) development of an embryo without the fertilization of the egg cell (parthenogenesis); and iii) endosperm development with (pseudogamous endosperm) or without (autonomous endosperm) fertilization of the central cell [48].

The most abundant GO terms found associated with the unigenes differentially expressed between sexual and apomictic accessions were those associated with transport, response to stress, biotic and abiotic stimuli, and DNA metabolic process and DNA binding (Figure 4). The number of enriched GO terms specifically related to plant reproduction (*i.e*., meiosis, gametogenesis, and embryonic and post-embryonic development) was relatively low in either sexual or apomictic genotypes (Figure 4). Nevertheless, our findings that unigenes found only in sexual or apomictic libraries were annotated as cell cycle, cell growth, cell differentiation, as well as embryonic and post-embryonic development (Figure 4) could provide some additional indications supporting the hypothesis that apomixis relies upon spatial or temporal mis-expression of genes acting during female sexual reproduction [27,30,49-52]. Hence a better understanding of the genetic mechanisms underpinning the switch from sexual to apomictic reproductive strategies could be greatly facilitated by the study of genes primarily involved in the normal progression of sporogenesis, gametogenesis and embryo development. In light of this, we examined whether apomictic seed development was characterized by expression by genes known to be important for the regular progression of gametogenesis and seed development in sexual plants.

One critical finding was the observation that gene products involved in cell differentiation and cell growth, as well as embryonic and post-embryonic development were overrepresented in the two sexual libraries (Figure 4). A closer expression analysis of multiple genes involved in meiosis (*HpDMC1*, *HpMS5*, *HpMND*) or megaspore selection (*HpAGP18*) indicates a relative decrease in mRNA abundance in apomictic pistils (Figure 6). The analysis of two distinct *HpDMC1*-like unigenes indicated that their respective mRNAs had contrasting expression patterns, and while *HpDMC1b* shows clear differential expression in all considered developmental stages of the pistil, the expression of *HpDMC1a* is similarly expressed in both apomictic and sexual pistils (Figure 6). Since cytological and cytometric data [19,29] indicate that apomictic plants in *H. perforatum* are characterized by a low occurrence of reduced embryo sacs, the decreased expression of critical genes for normal progression of meiosis supports the hypothesis that apospory in this species could be associated to a low rate of successful meiosis.

It has been hypothesized that the molecular switch from sex to apomixis is associated with gene-specific silencing mechanisms associated with chromatin remodeling factors or trans-acting and heterochromatic interfering RNAs involved in both transcriptional and post-transcriptional gene regulation [27]. Recently, a specific route for cell specification and embryo sac development was proposed to occur through the action of different *ARGONAUTE* (*AGO9* and *AGO5*) genes in *Arabidopsis* [13,14] and maize [15]. Similarly, mutations in *RDR6* or *SGS3,* two genes involved in the small RNA pathway, are known to result in a relaxation of gametic cell identity and fate in the ovule, leading to changes in cell fate of somatic initials in the nucellar tissue, which differentiate into gametic cells without undergoing meiosis and which can furthermore initiate female gametogenesis through the activation of TEs [14]. The RT-qPCR analyses performed here did not demonstrate differences in the abundance of RDR6 and SGS3 in sexual and apomictic pistils collected at developmental stages spanning female meiosis and gametogenesis (Figure 6). Similarly, no significant differences were detected in the expression of *HpAGO5* in the same plant tissues (Figure 7). Alternatively, since RT-qPCR analyses were performed on whole pistils, we cannot exclude the possibility that small expression differences consistent with gene expression in specific cell types of the ovule might have been lost. This could to be the case of *HpAGO5,* whose expression in *Arabidopsis* is expected in the nucellus, where it activates a non-cell autonomous mechanism by promoting the initiation of gametogenesis in the functional megaspore.

Our RT-qPCR data indicated that mRNA levels of *HpAGO9* are significantly decreased in apomictic pistils at the earlier developmental stages (Figure 6). Reduced expression of *HpAGO9* in pistils retaining a high level of apospory is noteworthy since *AGO9* is involved in restricting the acquisition of gametophyte cell fate in the sub-epidermal layer of the ovule nucellus in *Arabidopsis* [14], and the phenotype documented for *ago9 Arabidopsis* plants is remarkably similar to the aposporic gametophyte development [14].

Such considerations of the expression and activity of sRNAs and non-coding RNAs would support the idea that the coding fraction of the transcriptome could be only one side of the coin, and that small and non-coding RNA sequences could represent a meaningful component yet to be fully understood in the frame of gametes and seeds development.

## Conclusions

The availability of NGS technologies is raising the ease at which complex phenotypes and processes in non-model organisms can be analyzed as a consequence of the increased of throughput and availability of reference DNA and RNA datasets. In our study we were able to annotate and characterize 36,988 transcripts found expressed in male and/or female reproductive organs, including tissues or cells of sexual and apomictic flower buds. Our approach of sequencing whole flowers at different developmental stages from two fully obligate sexual genotypes and two unrelated highly apomictic genotypes, in addition to different flower parts dissected from a facultatively apomictic accession, enabled us to analyze the complexity of the flower transcriptome according to its main reproductive organs (*i.e*., sepals, petals, stamens and carpels) as well as for alternative reproductive behaviors (*i.e*., sexual *vs*. apomictic).

Our findings that unigenes found only in sexual or apomictic libraries were annotated as cell cycle, cell growth, cell differentiation, as well as embryonic and post-embryonic development provided additional indications supporting the hypothesis that apomixis relies upon spatial or temporal mis-expression of genes acting during female sexual reproduction. This hypothesis is further strengthened by our RT-qPCR data, demonstrating that genes expressed in sporophytic and gametophytic cell types and primarily involved in the normal progression of sporogenesis, gametogenesis and embryo development are differentially expressed between apomictic and sexual pistils.

Overall, the results collected in this research represent a critical step toward a better understanding of the genetic and molecular mechanisms leading to gametophyte and gamete determination and development in our model species, in either sexual or apomictic plant genotypes. Data presented here pave the way for whole transcriptome studies aimed at the identification and definition of transcriptome changes associated with the development of male and female reproductive organs by giving particular emphasis to meiosis, the formation of gametes and, more generally, to the processes associated with plant reproduction and seed formation in *H. perforatum*.

## Methods

### Plant materials

*Hypericum perforatum* L. plants from two naturally occurring tetraploids (2*n* = 4*×* = 32) and three induced tetraploids (2*n* = 4*×* = 32) were used for the sequencing approaches (Table 1). For the production of induced tetraploids, plants of the diploid sexual line R1, reselected from the tetraploid apomictic cultivar ‘Topaz’, were converted to auto-polyploids by colchicine application, as reported by Schallau *et al*. [18]. Briefly, seeds were imbibed in water for 24 h, placed on filter paper soaked with an aqueous solution of 0.2% colchicine for 24 h and then planted in soil for germination. The C0 plants that survived this treatment were self-pollinated and their progenies (C1) were screened for tetraploid plants. Sexual tetraploid C1 and C2 plants were used for further crosses with tetraploid apomictic pollinators and their progenies were then screened for the reproductive phenotype [18,29]. The reproductive mode of all *H. perforatum* accessions was estimated by flow cytometric screening of 48 single seeds as indicted by Matzk *et al*. [31].

We constructed a total of 8 different cDNA libraries. A single cDNA library was obtained from whole flowers collected across developmental stages 1-12 for each of the following accessions: HP13EU and HP36EU (sexual plant accessions), HP39EU and HP1973US (apomictic plant accessions). These four cDNA libraries were sequenced twice. An organ-specific cDNA library was also produced from the facultative apomictic accession HP4/13 for each of the following flower parts: buds (whole buds, length <3.0 mm, corresponding to *Arabidopsis* flower stages 1-10), carpels (bud length >3.0 mm, corresponding to *Arabidopsis* flower developmental stages 11-12), stamens (bud length >3.0 mm, corresponding to *Arabidopsis* flower developmental stages 11-12) and sepals/petals (bud length >3.0 mm, corresponding to *Arabidopsis* flower developmental stages 11-12) [18,53]. These four cDNA libraries were sequenced once.

### cDNA synthesis, normalization and pyrosequencing

Total RNA was extracted from whole flowers and flower parts by using the Spectrum™ Plant Total RNA Kit (Sigma-Aldrich) following the protocol provided by the manufacturer. The eventual contamination of genomic DNA was avoided by the optional DNase I (Sigma-Aldrich) treatment. Poly(A) + mRNA was isolated from total RNA using the Ambion MicroPoly(A) Purist Kit according to the manufacturer’s instructions (Life Technologies, Darmstadt, Germany) and the integrity and quantity was verified using an Agilent 2100 Bioanalyser and RNA Nanochips (Agilent Technologies, Santa Clara, USA). In order to avoid over representation of the most commonly transcribed genes and to maximize sequence diversity, normalized, full-length enriched cDNA libraries were generated using a combination of the SMART [54] cDNA library construction kit (Clontech, Takara, Saint-Germain-en-Laye, France) and the DSN normalization method [55] implemented in the Trimmer Direct cDNA normalization kit (Evrogen, BioCat, Heidelberg, Germany). The procedure generally followed the manufacturer’s protocol but included several important modifications, essentially as previously described in more detail [56]. Optimization of the complete cDNA normalization procedure, such as the number of thermocycles for ds-cDNA synthesis, was essentially carried out as described by Vogel and Wheat [57]. Each of the resulting ds-cDNA pools was purified and concentrated using the DNA Clean and Concentrator kit (Zymogen) and size fractionated with SizeSep 400 spun columns (GE Healthcare) that resulted in a cutoff at ~200 bp. To test for normalization efficiency, a fraction of the full-length-enriched cDNAs were cut with *Sfi*I and ligated to pDNR-Lib plasmid (Clontech). Ligations were transformed into *E. coli* ELECTROMAX DH5α-E electro-competent cells (Invitrogen). For each cDNA library, appr. 200 bacterial colonies were grown in 96 deep-well plates, plasmid mini-preparations were performed using the 96well robot plasmid isolation kit (NextTec) on a Tecan Evo Freedom 150 robotic platform (Tecan) and Single-pass sequencing of the 5’ termini of cDNA libraries was carried out on an ABI 3730 xl automatic DNA sequencer (PE Applied Biosystems). Vector clipping, quality trimming and sequence assembly using stringent conditions (*e.g*., high quality sequence trimming parameters, 96% sequence identity cutoff, 35bp overlap) was done with the Lasergene software package (DNAStar Inc.). For direct sequencing, approximately 1 μg of each *Sfi*I-digested normalized cDNA sample was sheared via nebulization into small fragments. The eight shotgun cDNA libraries were grouped in two plates and sequenced on the Roche 454 FLX platform using Titanium chemistry (Roche Diagnostics Corporation, Basel, Switzerland).

### Bioinformatics - EST processing protocol

All high quality reads generated from the two sequencing reactions were assembled in a single reference transcriptome. The assembly was done with gsAssembler (Newbler v2.7, Roche Diagnostics Corporation, Basel, Switzerland) by considering the default parameters, including a minimum read length: 20; seed length: 16; seed step: 12; minimum overlap length: 40 and minimum overlap identity: 90. Both contigs and singletons originated from this procedure were then used as input files in a second assembly step in which all parameters were maintained equal to the previous assembly, except that the minimum overlap length was lowered to 20 bp.

For each single library and assembled sequence, the relative abundance of predicted unigenes was calculated by counting the number of raw reads taking part in the assembly. A unigene was considered expressed in one library if at least one sequence derived form the specified library was included in the assembly of the considered unigene. Transcriptome overlaps and compositions were displayed with Venn diagrams using the web tool venny (bioinfogp.cnb.csic.es/tools/venny/index.html; [58]).

A BLASTX-based approach was used to compare the *Hypericum* sequences to the nr database and to annotate the assembled *Hypericum* unigenes, using the BLAST v2.2.29+ downloaded from NCBI, National Center for Biotechnology Information (http://www.ncbi.nlm.nih.gov/).

The *H. perforatum* clone BAC 25H09 genomic sequence containing the apospory-linked marker and covering the apospory-associated region (GenBank accession number HM061166.1) was previously identified and annotated for several predicted genes, including a variety of transcription factors and retrotransposons [18]. Alignments of unigenes to the BAC sequence HM061166.1 were performed with software the CLC Genomics Workbench v7 (QIAGEN). Statistics on genetic diversity and distribution of the average number of nucleotide substitutions per site (Dxy) were performed with the software DNAsp v5.10.1 [59]. Values of Dxy were extracted from the package of statistics: DNA divergence between populations (sliding window: 100 bp; overlaps: 25 bp).

To annotate all assembled unigenes (contigs and singletons), a BLASTX-based approach was used to compare the *Hypericum* sequences to the nr database downloaded from the NCBI (http://www.ncbi.nlm.nih.gov/). Moreover, the GI identifiers of the best BLASTX hits, having E-value ≤ 1 E-09 and similarity ≥ 70%, were mapped to UniprotKB protein database (http://www.uniprot.org/) in order to extract Gene Ontology (GO, http://www.geneontology.org/) and KEGG orthology (KO, http://www.genome.jp/kegg/) terms for further functional annotations. The BLAST2GO software v1.3.3 (http://www.BLAST2go.com; [60]) was used to reduce the dataset to the GOslim level (goslim_plant.obo) and perform basic statistics on ontological annotations, as reported by [61].

### Plant reproduction database

The plant reproduction database was generated based on the refseq protein sequence repository at GenBank that could be downloaded by browsing the dataset with opportune key words such as (“embryo defective”[All Fields], “maternal effect embryo arrest”[All Fields], “unfertilized embryo sac”[All Fields], “gametophytic factor”[All Fields]). Searches were restricted to the Plants by using the option “Viridiplantae”[Organism]. In a parallel approach, all sequences annotated as “meiosis”, “gametophyte development”, and “embryogenesis” were downloaded from TAIR (http://www.arabidopsis.org/). Finally, a number of protein sequences whose gene products were studied and linked to “cell specification”, “meiosis”, “gametogenesis” and “seed development” [3-5,10-12] were downloaded from Genbank and added to the previous set. On the whole, the plant reproduction database considered 811 non-redundant *Arabidopsis* gene loci.

### Validation of sequencing data by quantitative Real-Time RT-PCRs

Plant materials were selected according to the genetic and cyto-histological bases of apospory recently described for *Hypericum perforatum* [18,19]. The reproductive mode of all *H. perforatum* accessions was estimated by flow cytometric screening of 48 single seeds as indicted by Matzk *et al*. [31]. Samples were collected in 3 biological replicates, from different plant accessions (Table 1). RNA extractions were conducted using the Invisorb Spin Plant RNA Mini Kit (Sigma-Aldrich). cDNA synthesis was conducted using the SuperScript® VILO™ cDNA Synthesis Kit (life technologies), following the indication of the supplier. Primers used in the quatitative RT-PCR experiments are reported on Additional file 1: Table S8. Expression analyses were performed using the thermal cyclers StepOne and 7300 Real-Time PCR System (Applied Biosystem), equipped with 96- and 384-well plate systems, respectively, with the SYBR green PCR Master Mix reagent (Applied Biosystem). The amplification efficiency was calculated from raw data using the OneStep Analysis software (Life Technologies). Amplification performances expressed as fold change were calculated by the ΔΔCt method using *HpTIP4* as housekeeping gene [62]. Error bars indicate the standard deviation observed among the three biological replicates.

### Computational investigation of flower and seed-related genes based on *Arabidopsis* microarray-based data

An *Arabidopsis* microarray-based reference transcriptome was created using the *Arabidopsis*-expressed sequences, downloaded from publically available experiments. Microarray data sets were downloaded from the arrayexpress data repository at EBI (http://www.ebi.ac.uk/arrayexpress/experiments/) and used to infer the most probable *Arabidopsis* transcriptome to be used as a reference (Additional file 1: Table S7). Probes were considered expressed when present in at least one experiment, considering a p-value lower than 0.05.

MapMan (http://mapman.gabipd.org/web/guest/mapman; [63]) analyses were performed by using the *Hypericum* dataset properly rearranged as input files. Briefly, the *Arabidopsis* proteome was downloaded from the TAIR, The Arabidopsis Information Resource database (ftp://ftp.arabidopsis.org/home/tair/Sequences/BLAST_datasets/TAIR10_BLASTsets/) and set as reference database for local BLASTX analysis performed using the *H. perforatum* transcriptome dataset as the query. AGI codes relative to *Arabidopsis* putative homologous genes (E-value cut-off: 1.0E-9) were recovered by each tabular BLASTX result (BLAST-2.2.25+ argument: -outfmt 6) and used to download the corresponding ATH1-121501 genechip identifiers (Affymetrix) from TAIR (http://www.arabidopsis.org/tools/bulk/microarray/index.jsp) (for more details see [61]). MapMan analyses focused on maps: Regulation overview and Cellular response overview, which were used to graphically display the data.

### Availability of supporting data

Raw sequences files were made available for download from SRA with accession numbers: SRR1646951, SRR1646953, SRR1646955, SRR1646956, SRR1647632, SRR1647633, SRR1647673, SRR1647674, SRR1647677, SRR1647678, SRR1647713, SRR1647714. Sequences of unigenes investigated by Real-Time RT-qPCR along with unigenes aligned to the BAC sequence HM061166.1 were deposited as Transcriptome Shotgun Assembly project, which has been deposited at DDBJ/EMBL/GenBank under the accession GBXG00000000. The version described in this paper is the first version, GBXG01000000.

## Additional files

Additional file 1: Table S1. Descriptive statistics of the sequenced *Hypericum* flower libraries. **Table S2.** Main statistics concerning the BLASTX sequence comparisons. **Table S3.** BLAST matches produced by comparing the assembled unigenes with the CDS deduced from the *Hypericum* BAC clone HM061166.1. **Table S4.** Abundance of unigene sequences matching the *Hypericum* BAC clone HM061166.1. **Table S5.** Ontological annotations ascribed to the *Hypericum* flower transcripts. **Table S6.** *Hypericum* unigenes that show high similarity with *Arabidopsis* genes annotated for terms related to plant reproduction *sensu lato*. **Table S7.** Publically available *Arabidopsis* microarray experiments used for comparisons with *Hypericum* flower transcriptomes. **Table S8.** Primers used in all Real-Time RT-PCR experiments (for additional information on each table, please see details reported in the additional file itself). Additional file 2: Figure S1. Genetic diversity computed for the unigenes aligning to the *Hypericum* BAC clone HM06166. Alignments of multiple unigenes to the sequence extracted from HM61166.1 and the corresponding predicted CDS. Nine genes producing alignments with multiple unigenes are shown. For each alignment, the distribution of the average number of nucleotide substitutions per site (Dxy) calculated between unigenes and the aligned region of HM61166.1 is shown. Additional file 3: Figure S2.
*Hypericum* unigenes matching *Arabidopsis* flower and seed transcripts. Venn diagrams that graphically represent the distribution of unigenes matching an *Arabidopsis* gene model included on the *Arabidopsis* microarray-based reference transcriptome. A: main developmental stages (A: fertilized flowers, non fertilized flowers) considered in the *Arabidopsis* microarray-based reference transcriptome. B: main flower and seed organs included on the *Arabidopsis* microarray-based reference transcriptome. Additional file 4: Figure S3. Overview of *Hypericum* transcripts involved in regulation and cellular response processes. Graphical representation of the *Hypericum* flower and seed transcriptomes produced with MapMan. Blue squares indicate significant matches with genes expressed in the *Arabidopsis* flower. Light blue: match within the flower, but found only in apomictic libraries; green: match within the flower, but found only in sexual libraries. Red squares indicate specific matches in the *Arabidopsis* seed dataset. Violet: seed library and found only in apomictic libraries; Orange: seed library and found only in sexual libraries. Black: sequences that could not find a match in the flower or seed reference databases.

## Abbreviations

BAC: Bacterial artificial chromosome
BLAST: Basic local alignment search tool
CDS: Coding sequence
HAPPY: Hypericum APOSPORY
ITS: Internal transcribed spacers
NCBI: National center for biotechnology information
NGS: Next generation sequencing
GO: Gene ontology
RPK: Number of reads per kilobase
RT-qPCR: Reverse transcription quantitative polymerase chain reaction
SNP: Single nucleotide polymorphism
TAIR: The arabidopsis information resource
