# Supplementary figures and images for: De novo sequencing of the Hypericum perforatum L. flower transcriptome to identify potential genes that are related to plant reproduction sensu lato
Source: BMC Genomics. 2015 Mar 31;16(1):254. doi: 10.1186/s12864-015-1439-y (PMC4451943; doi:10.1186/s12864-015-1439-y)

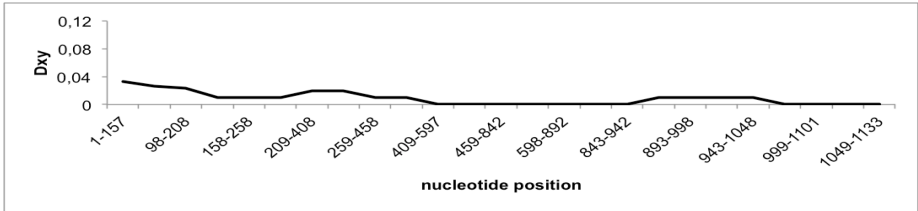

T1 - Exp1

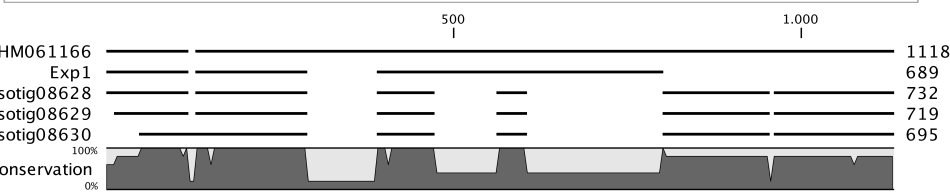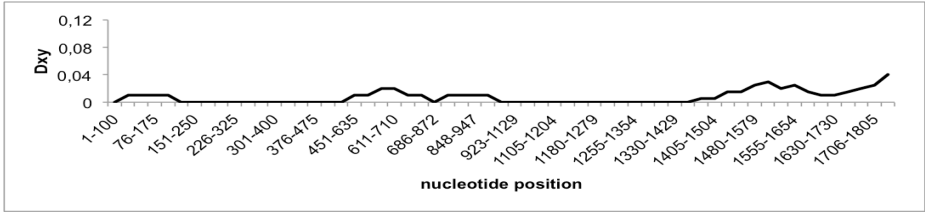

T2 - WRKY

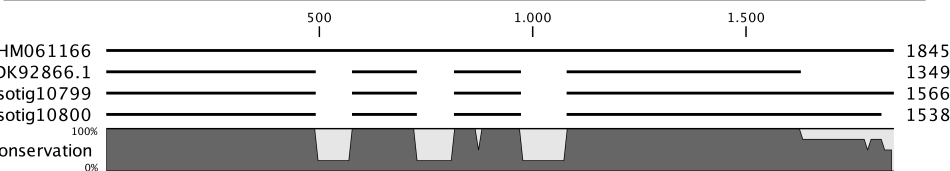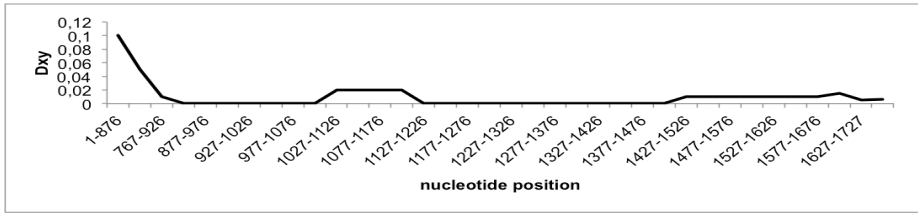

T3 - DGCR

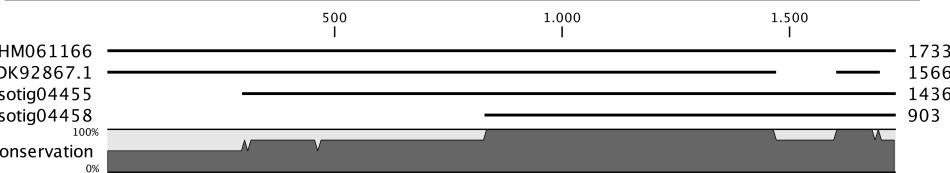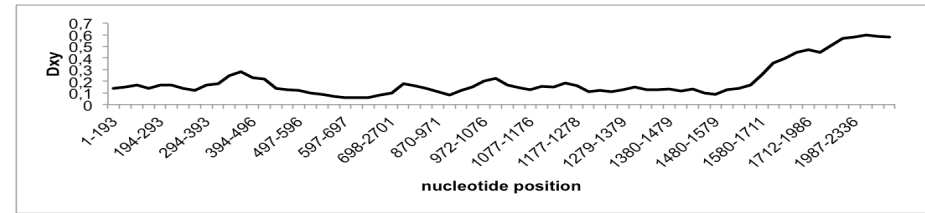

T4 – ARI7

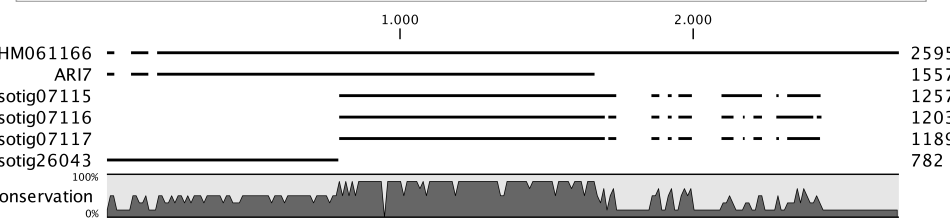

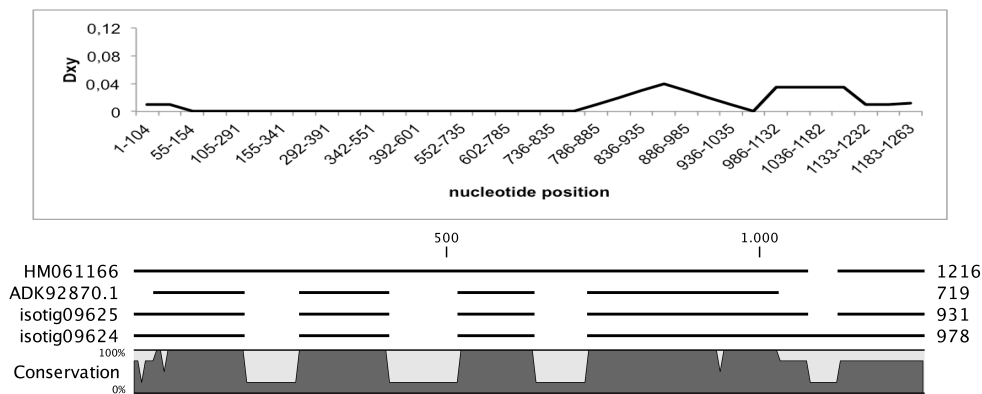

T5 - TK

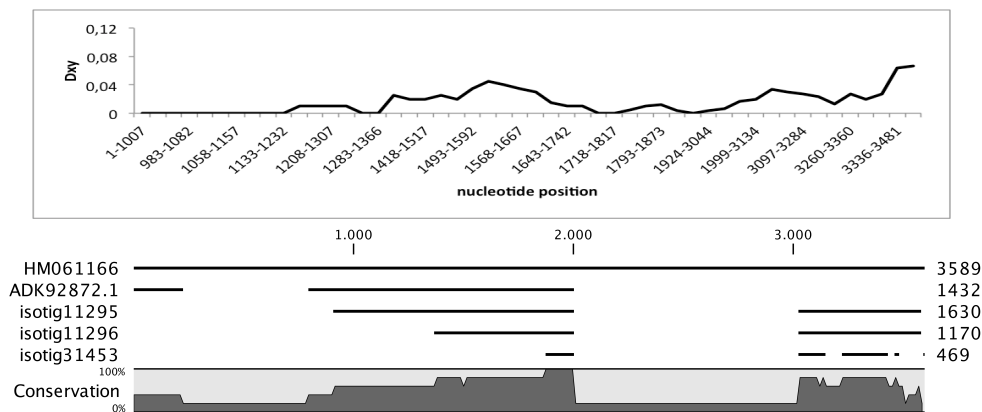

T6 - HnRNP

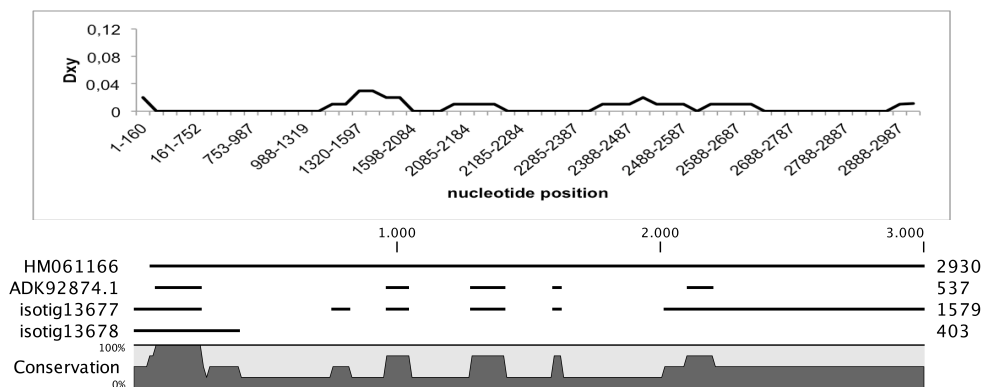

T7 - RimL

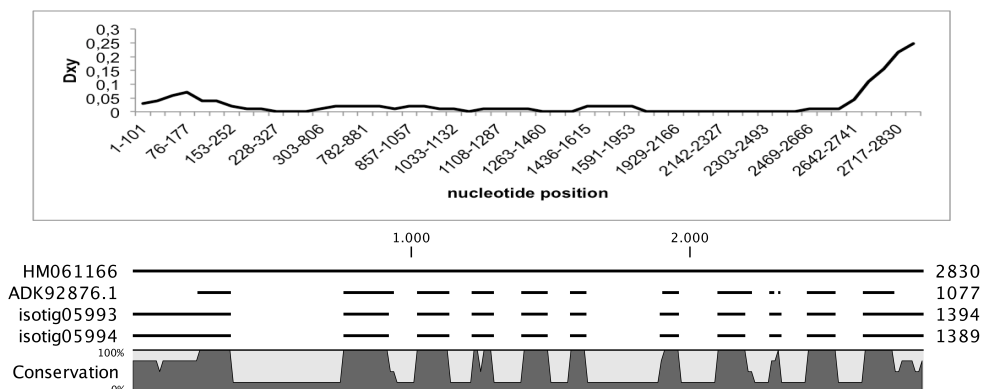

T8 - Mo25

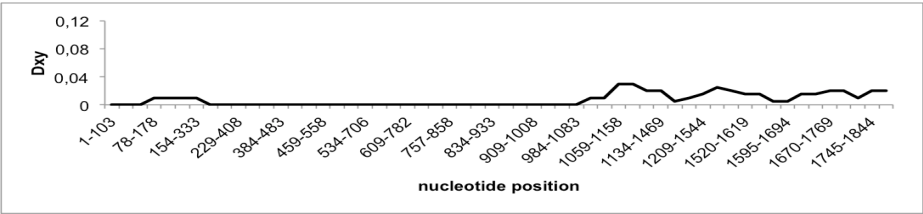

T9 - PG

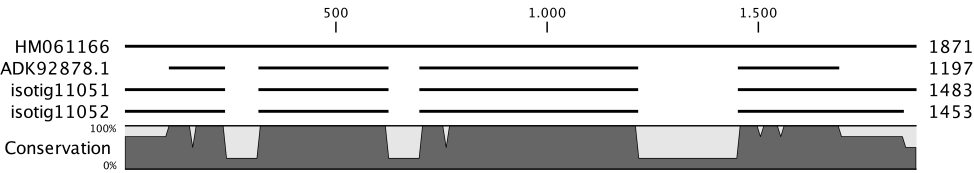

Supplement: Additional file 2: Figure S1. — Genetic diversity computed for the unigenes aligning to the Hypericum BAC clone HM06166. Alignments of multiple unigenes to the sequence extracted from HM61166.1 and the corresponding predicted CDS. Nine genes producing alignments with multiple unigenes are shown. For each alignment, the distribution of the average number of nucleotide substitutions per site (Dxy) calculated between unigenes and the aligned region of HM61166.1 is shown. [file 12864_2015_1439_MOESM2_ESM.pdf]

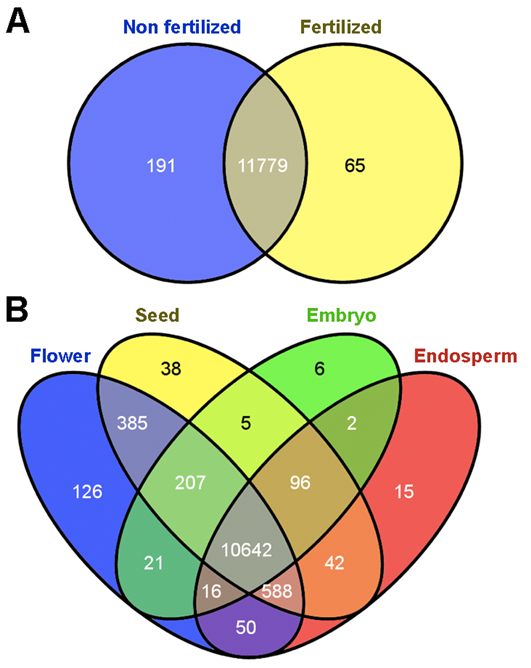

Supplement: Additional file 3: Figure S2. — Hypericum unigenes matching Arabidopsis flower and seed transcripts. Venn diagrams that graphically represent the distribution of unigenes matching an Arabidopsis gene model included on the Arabidopsis microarray-based reference transcriptome. A: main developmental stages (A: fertilized flowers, non fertilized flowers) considered in the Arabidopsis microarray-based reference transcriptome. B: main flower and seed organs included on the Arabidopsis microarray-based reference transcriptome. [file 12864_2015_1439_MOESM3_ESM.tiff]

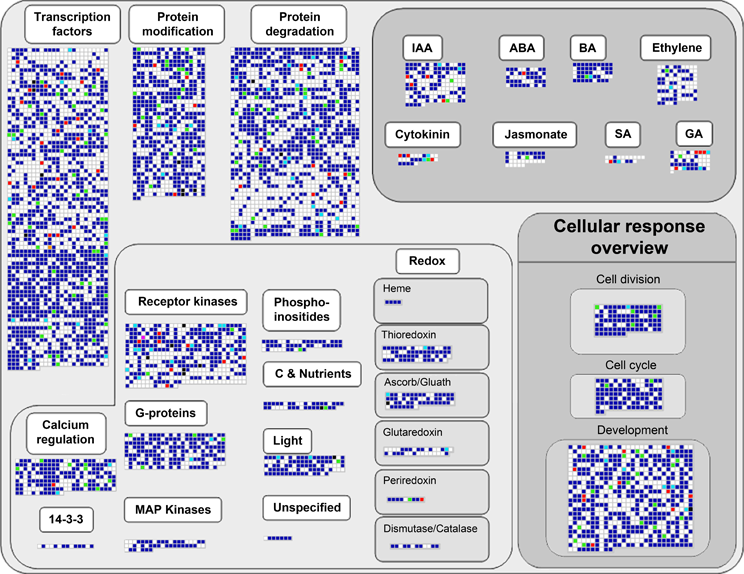

Supplement: Additional file 4: Figure S3. — Overview of Hypericum transcripts involved in regulation and cellular response processes. Graphical representation of the Hypericum flower and seed transcriptomes produced with MapMan. Blue squares indicate significant matches with genes expressed in the Arabidopsis flower. Light blue: match within the flower, but found only in apomictic libraries; green: match within the flower, but found only in sexual libraries. Red squares indicate specific matches in the Arabidopsis seed dataset. Violet: seed library and found only in apomictic libraries; Orange: seed library and found only in sexual libraries. Black: sequences that could not find a match in the flower or seed reference databases. [file 12864_2015_1439_MOESM4_ESM.tiff]
